# Range of dose enhancement factor for gold nanoparticles in radiotherapy using Monte Carlo simulations

**DOI:** 10.1007/s00411-026-01245-4

**Published:** 2026-08-08

**Authors:** Wei Bo Li, Mario A. Bernal, Ziad Francis

**Affiliations:** 1 Institute of Radiation Medicine, Helmholtz Zentrum München, German Research Center for Environmental Health (GmbH), Ingolstädter Landstraße 1, 85764 Neuherberg, Germany; 2 Federal Office for Radiation Protection (BfS), Ingolstädter Landstraße 1, 85764 Oberschleißheim, Germany; 3 Instituto de Fisica Gleb Wataghin, Universidade Estadual de Campinas, Campinas, 13083- 859 SP Brazil; 4 Research Unit Mathematics and Modeling, Saint Joseph University of Beirut, Beirut, 1004, 2020 Lebanon

**Keywords:** Gold nanoparticle, Radiation, Dose enhancement factor, Radiotherapy, Monte Carlo simulation

## Abstract

Gold nanoparticles (GNPs) have been widely investigated as radiosensitizers in preclinical radiotherapy studies and are now advancing into clinical trials. To enhance tumour targeting, various molecular vectors are conjugated with GNPs. The localized radiation energy deposited near GNPs mainly originates from photoelectrons, Auger electrons, x-ray fluorescence, and Compton electrons, resulting in higher tumour doses compared to conventional radiotherapy. Due to the nanometer-scale size of GNPs, the energy from secondary low-energy electrons is confined to nanometer to micrometer ranges, making direct dose measurements impractical. Monte Carlo (MC) simulations are therefore commonly employed to estimate absorbed dose distributions around GNPs under different experimental conditions. However, published MC results show significant variability due to diverse simulation parameters. This review presents a quantitative analysis of reported dose distributions and dose enhancement factors (DEFs) from MC simulations. It also discusses the range of physical radiation doses and DEFs for GNPs irradiated by various radiation types, including kV/MV x-rays, synchrotrons, electrons, protons, heavy ions, and therapeutic radionuclides. To support understanding of the dose enhancement mechanisms, the underlying physical interaction processes of these radiation types with water and gold are reviewed. The range of reported DEFs helps bridge the gap between physical dose enhancement and observed biological effects. Finally, we recommend a qualitative estimate of the contribution of chemical species produced by the GNPs interactions to the consequent enhanced biological effects. This allows total dose enhancement from GNPs in radiotherapy, considering physical and chemical aspects, to be estimated using MC simulations as a complement to experiments.

**Supplementary Information:**

The online version contains supplementary material available at https://doi.org/10.1007/s00411-026-01245-4.

## Introduction

Gold nanoparticles (GNPs), with a high atomic number (high-Z) of 79, have been investigated as contrast agents, photothermal agents, drug carriers, and radiosensitizers for many years (Jain et al. 2012). GNPs are nanometer-scale particles made of gold with different shapes and sizes, for example in the forms of spheres, rods, shells, stars, cages, boxes, and prisms, in the sizes from 2 nm to 500 nm (Panchapakesan et al. 2011). In the application of ionising radiation for cancer treatment, the ultimate goal is to keep or even enhance the therapeutic dose to the tumour and to reduce the radiation dose in normal surrounding tissues at the same time, so that the risk of developing toxic side effects can be reduced. In searching for a greater therapeutic efficacy in targeted radiotherapy (Ngwa et al. 2014), earlier works using gold foils (Regulla et al. 1998) and gold microspheres (Herold et al. 2000) demonstrated the enhancement of the physical dose and biological effects around the interface of gold and soft issues. The pioneering work of Hainfeld et al. (2004) with intravenous injection of GNPs targeting tumour in mice irradiated by 250 kV x-rays firstly demonstrated the enhancement of the therapeutic efficacy using nanoparticles of gold. Following this novel experimental work, Cho (2005) proposed a theoretical Monte Carlo (MC) simulation to estimate radiation dose from secondary electrons around the GNPs irradiated by x-rays. Thereafter, in vivo and in vitro preclinical investigations with GNPs in different radiotherapy settings boomed (Chithrani et al. 2010; Yang et al. 2014; Chattopadhyay et al. 2013; Mueller et al. 2020; Berbeco et al. 2011; Zhang et al. 2021; Jain et al. 2011; Kim et al. 2012). Simultaneously, theoretical simulation works using various MC codes, for example, PENELOPE, Geant4/Geant4-DNA, TOPAS/TOPAS-nBio, PARTRAC, NASIC, MDM, TRAX, EGSnrc, MCNP6 and more have been improved and extended implementing various preclinical geometrical setups and different radiation types, such as kilovoltage (kV), megavoltage (MV), and synchrotron photons (McMahon et al. 2008; Cho et al. 2009; Jones et al. 2010; Leung et al. 2011; Lechtman et al. 2011; Douglass et al. 2013; Zygmanski et al. 2013; Xie et al. 2015), electrons, protons, heavy ions, and several radionuclides among more further references which will later be analysed in this review. As preclinical experimental investigation with GNPs has been increasing, understanding the detailed mechanisms behind dose and biological enhancement effects was highly desired, for example from the physical radiation interactions (Kuncic and Lacombe 2018), the physicochemical and radiochemical interactions (Sicard-Roselli et al. 2014; Gilles et al. 2018; Guo 2019), and the biological processes (Rosa et al. 2017). Due to their presumed biocompatibility, strong photoelectric absorption, and the generation of x-ray fluorescence as well as Auger and Coster–Kronig (C–K) electrons upon irradiation, GNPs have been extensively investigated for decades as possible agents for a selective amplification of the radiation dose in tumours. In 2016, Schuemann et al. (2016) outlined a roadmap to the clinical use of GNPs for this radiation sensitization. Four years later, a second roadmap for more metal nanoparticles was outlined and covered the current status, translation challenges, and further directions (Schuemann et al. 2020).

In this context, theoretical quantification of the GNP-induced dose enhancement is needed not only for difficulties of measuring the dose in the GNP vicinity, from the nanometer to micrometer ranges, but also for establishing a dose-effect relationship for this novel GNP-assisted radiation therapy (Cooper et al. 2014; Ngwa et al. 2014; Zygmanski and Sajo 2016). Determination of the enhanced dose is generally done using MC codes for radiation transport simulation. However, the simulation results in the literature showed large differences due to various factors, such as geometrical setup and the scale where energies deposited are scored, macroscopic or microscopic. In some cases, a MC code allows using different physical models for cross section determination so that different dose distributions could be obtained even using the same code. The objective of this paper is to review and summarise works on the determination of the dose enhancement factor (DEF) around GNPs, using MC simulations, covering a wide range of combinations of incoming particles and their energy spectra, geometrical setups, GNP sizes, and Monte Carlo codes. We may have a picture on how all these factors and the associated radiation-matter interaction processes influence the dose enhancement. Furthermore, we can estimate the contribution of chemical radicals and biological effects to the biological enhancement effect in general. As the dose enhancement is extremely localised around the GNP, a selective and relatively intense uptake of GNPs into tumour cells is required. To reach a high concentration of GNPs inside cancer cells, a functionalized GNP with a targeting antibody or other coating material may be used to transfer GNPs into the cells, cytoplasm, or even to be internalised in the cell nucleus. However, in this review, we will not estimate the effects of the coating vectors on the GNP dose enhancement. Consequently, this review aims to elucidate and provide the physical dose enhancement effects and contribution of GNPs with MC simulation approaches, thereby facilitating their transitioning toward clinical trials in radiotherapy.

### Scope of the review

In this review, we will first provide an overview in Sect. 2 on the most relevant interaction processes in the present context, when photons, electrons, protons, and heavy ions interact with liquid water and gold. Sect. 3 presents a short overview on the application of MC simulations in irradiating GNPs. In Sect. 4, we will address the dose enhancement due to the presence of GNP, grouping reviewed articles according to the incoming radiation type, then they will be indicated as single and multiple GNP distributions. In vitro and in vivo environments will be included in the discussion. In Sect. 5, we will discuss the review results and perspective on the further work in GNP enhanced radiation therapy. In the last Sect. 6, we draw main conclusions from our review and provide recommendations. In the Supplementary Information, we provide the fundamentals of physical interactions of radiation with liquid water and gold in S2.1 and thorough information on GNP dose enhancement in MC simulation in a nutshell table S2.

### Definition of dose enhancement factor

**Fig. 1:**
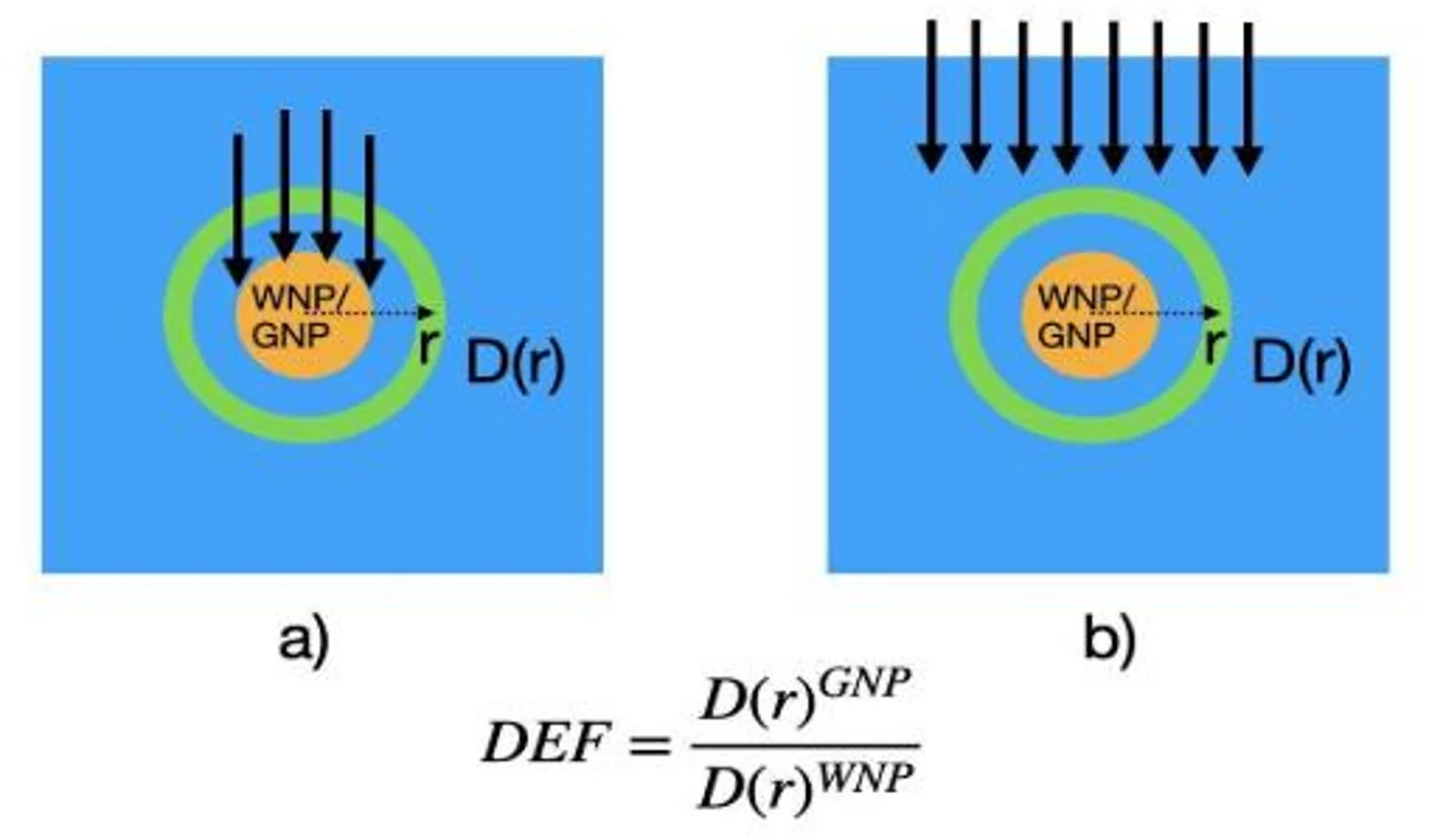
Most commonly used irradiation setup when the DEF is determined by Monte Carlo simulations

The Dose Enhancement Factor (DEF) is defined for quantifying the increase of the absorbed dose around the GNP when compared with the situation in which there is only tissue or water in the irradiated medium. Figure 1 shows the irradiation setup for a single GNP and the corresponding DEF definition. The DEF is the ratio between the doses at the volumes investigated, for example a shell around the nanoparticle with the GNP in place to that with the corresponding water nanoparticle (WNP). Figure 1 shows two commonly used configurations when determining the DEF for a single GNP. Notice that the DEF is defined as the ratio between the doses with and without the presence of the GNP at a given distance from the particle. These setups implicitly considered that the dose distribution follows a spherical symmetry around the GNP, which may not be accurate if primary as well as secondary particles are accounted for in simulations. In the configuration a), only the GNP is irradiated by the incoming particles and only secondary electrons produced by their interaction with the GNP, are accounted for in the absorbed dose determination. The motivation of this configuration is considerations of computational efficiency; however, it has a deficiency of charged particle equilibrium. In the setup b), the beam covers a cross-section area larger than that defined by the GNP itself. Some authors (Wälzlein et al. 2014; Heuskin et al. 2017) only account for the secondary radiation emitted by the GNP while others (Martínez-Rovira and Prezado 2015) also include the primary incoming particles. The advantage of this setup is the solution of the charged particle equilibrium, but it increases the computation times. At first glance, it can be stated that the macroscopic enhancement of the dose distribution would depend on the GNP density and the range of the microscopic dose enhancement around each of them. The lower the range of the secondary electrons produced by the radiation-GNP interaction, the higher the GNP concentration that must be used for obtaining an appreciable increase in the macroscopic dose distribution, and vice-versa. Other authors (Peukert et al. 2020b; Heuskin et al. 2017) studied the dose enhancement phenomenon in a multi-GNP scenario. This means that the DEF is determined for a given distribution of GNP, characterized by a certain concentration. Therefore, a third setup for defining the DEF is that in which gold is regarded as totally diluted in water, at a given concentration, rather than explicitly simulating GNP (Cho et al. 2016). Based on these observations, DEF determination can be classified according to the determination setup, as follows: Setup I - A single GNP is irradiated with a narrow beam, large enough to just cover the GNP cross section. The simulation is repeated by replacing the GNP with a water nanoparticle (WNP). The ratio between the dose at a given point with GNP and with the WNP are defined as the DEF. Setup II - A cluster of GNPs embedded in water is irradiated with a beam large enough to cover the distribution region. The simulation is repeated with only water and the ratio of the dose at a given point with and without the GNPs are defined as the DEF. Setup III - A gold aqueous solution with a given weight concentration is irradiated and the corresponding dose is compared to that obtained in pure water at the same location. In all cases, DEF will be defined as the ratio of the doses at a given point with and without gold present in the irradiated volume.

## Radiation interactions

**Fig. 2:**
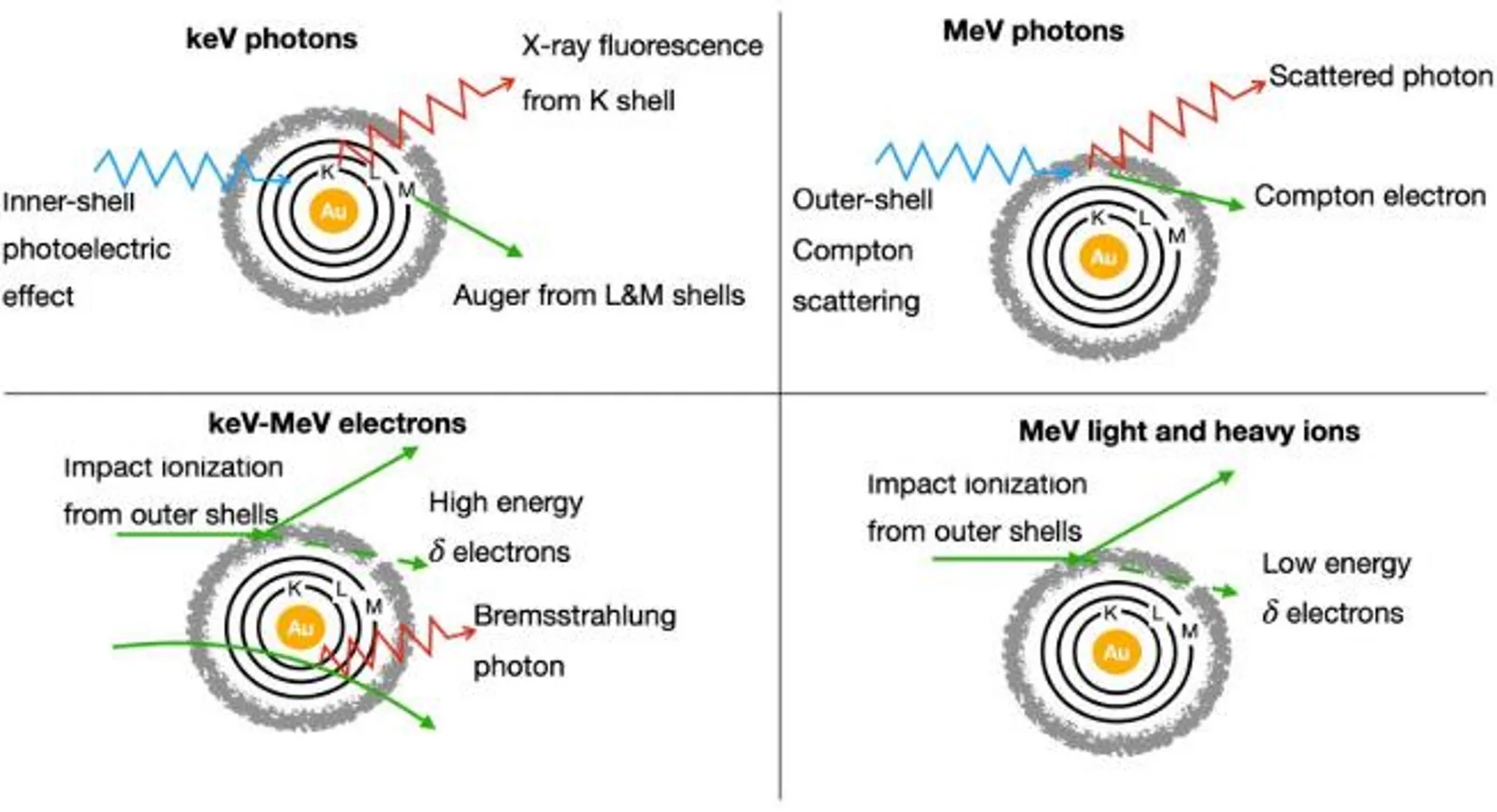
Sketch with the most important physical processes for the problem in question within this review, according to the incoming particle and its energy range. It is important to note that charged particles can also produce inner shell ionizations, albeit with a much lower probability than in valence shells. In addition, Bremsstrahlung emission at MeV energies for ions is negligible. Furthermore, electrons can transfer a greater fraction of their energy when compared with ions. Low energy photons preferentially undergo photoelectric effect in inner shells while high-energy photons produce Compton scattering off valence electrons most of the time

In this section, the fundamentals of physical interactions of radiation with liquid water and gold were summarised in Fig. 2. Furthermore, we provide the fundamentals of interaction of radiation with gold and water, and the main mechanisms for the contribution to the enhancement of the radiation dose in the supplementary material.

## Monte Carlo simulation in GNP radiation

Experimental determination of DEF is extremely difficult because of the small spatial scales involved in this problem. Furthermore, dose distribution determination at small scales demands considering very low energy electrons, which are the primary particles responsible for dose deposition regardless of the incident radiation. They have extremely short ranges so even thin foils can stop them and they cannot reach the detector. For these reasons, it is challenging to resolve these dose distributions experimentally. Thus, Monte Carlo simulation emerges as a natural tool for studying this problem.

There are some basic requirements for a MC code to be used in this kind of study. It must handle water and gold as transport media. This is fulfilled for virtually all general-purpose MC codes. Another key question is the capacity of the MC code to track electrons down to very low energies since a high spatial resolution is mandatory in these applications. The reader should be aware that the range of secondary electrons at the transport cut-off energy should be shorter than the characteristic spatial resolution. In addition, the code should be able to discriminate between the different processes that lead to secondary electron production, like impact ionisation, photoelectric effect, Compton scattering, pair production, and Auger emission. In principle, this discrimination allows to resolve the importance of each of these processes in the dose enhancement due to the GNP presence. Several MC codes can be used in this field, such as PENELOPE (Salvat et al. 2012), MCNP (Werner et al. 2018), Geant4 (Agostinelli et al. 2003), and EGSnrc (Kawrakow 2000; NRC 2021). Up to our knowledge, all MC codes available for medical applications treat all media as bulk material when determining interaction cross sections. However, it should be noted that surface effects may arise when reducing the amount of gold down to nanometric dimensions.

From the geometrical perspective, GNP can be simulated as a single particle or as nanoparticles distribution defined for a given GNP density, measured as the mass of gold per unit water mass. Simulations with a single GNP can give us an idea on how the dose distribution is affected by the particle, at very short distances from it. However, the influence of GNP on the macroscopic dose can be only determined by accounting for a GNP volumetric distribution. It is also important to remark that GNP size influences dose distribution in the vicinity of the particle by modulating the x-ray interaction with gold and self-absorption of secondary particles, like Auger electrons and fluorescence x-rays.

When dealing with primary photon beams, incoming photons can be treated as mono-energetic or coming from complex spectra, as in the case of x-rays, including MV beams. Charged particle beams are commonly modelled as mono-energetic. Simulating the whole process for producing x-ray beams is very expensive from the computational point of view. Then, simulations generally start from spectra determined by some package available in the market, like *SpekCalc* for kV beams (Poludniowski et al. 2009). MV spectra are commonly obtained from Monte Carlo simulations (Sheikh-Bagheri and Rogers 2002).

## Energy deposition and dose enhancement factor

### Photons

#### Photons in kV and MV energy

Here, we summarize the energy deposition and DEF for a single GNP in bulk water and for multiple GNPs in a cellular environment irradiated by photons. According to setups I-III discussed and defined in Sect. 1.2, the DEF can vary significantly depending on the presence of single or multiple GNPs and irradiation geometries implemented. Many investigators simulated various scenarios within a single publication, therefore throughout the review, keywords, like *single*,* multiple*,* compound* and *cluster* are provided alongside the citations to clearly identify the corresponding radiation scenarios. The studies were in general reviewed chronologically, however, works involving similar simulation setups were grouped together where being appropriate to facilitate comparison, analysis, and synthesis of the findings. Additional details can be found in Table S2 of the Supplementary Information.

MC simulations of GNPs irradiated by photons in the early years mainly focused on single GNPs under simple geometrical conditions, for example, the sampled photon sources were of similar size to the GNP diameters without considering the lateral secondary particle disequilibrium. Later on, the enhancement effects of GNPs in multi-particle configurations (compounds and clusters) were also included in the studies, with the aim of better predicting experimentally observed effects. Cho (2005) (*compound*) may have been the first to use Monte Carlo simulations (BEAMnrc/DOSXYZnrc) to study GNP dose enhancement in a tumour within a tissue phantom, irradiated by 140 kVp x-rays, 4 and 6 MV Linac photon beams, and ^192^Ir gamma rays. A DEF of at least 2 was found for 140 kVp x-rays at 7 mg Au g^− 1^, while 4–6 MV photon beams had DEFs of 1.01–1.07 and ^192^Ir ranged from 1.05 to 1.31. Gold was treated as a uniform mass rather than nanoparticles, calculating DEF macroscopically. The study emphasized considering microscopic dose enhancement, referencing Regulla et al. (1998), which found up to 100-fold physical and 50-fold biological dose increases with gold. It advocated for event-by-event Monte Carlo simulations for detailed electron track modelling. In the next few years, similar investigations were performed. Carter et al. (2007) (*single*) calculated energy deposition around 3 nm GNPs using a 100 kVp tungsten x-ray spectrum (Tucker et al. 1991). Energy deposition ranged from 10^− 5^ to 10^− 9^ eV nm^− 3^ at 1–100 nm from the GNP and 10^− 6^ to 10^− 9^ eV nm^− 3^ at 1–20 nm from secondary electrons in water. Without GNPs, energy deposition in water was 9 × 10^− 6^ eV nm^− 3^. The DEF was very low when assessing energy deposition in a 1 nm water shell around the GNP. In the work (Zhang et al. 2009) (*multiple*), using Geant4, GNPs in dimeter of 100 nm were placed in a 10 × 10 × 10 mm³ water phantom irradiated by a ^192^Ir HDR source 10 mm above. A parallel beam showed dose enhancement, which increased to 60% with a 4π-isotropic beam due to reduced lateral electronic equilibrium. No DEF was simulated at nanometer scale. Jones et al. (2010) (*compound*) studied secondary electron energy deposition from GNPs at a nanometer scale using six photon sources (^125^I, ^103^Pd, ^169^Yb, ^192^Ir, 550 kVp, 6 MV). Tumours with 7 mg Au g^− 1^ showed up to two orders of magnitude higher electron fluence, with DEFs of 2–20 within 5 μm and 1.05 up to 30 μm. The study supported previous gold foil findings but treated gold as a mass material, estimating DEF via dose point kernels rather than direct GNP-water simulations.

Around 2011, several studies examined MC simulated DEF using different GNP geometries. Leung et al. (2011) (*single*) used Geant4 to simulate electron tracking from 2, 50, and 100 nm GNPs irradiated by x-rays in water. GNPs increased secondary electron production by 10–2000 times, with low-energy beams interacting more efficiently than MV energies. Larger GNPs contributed more to dose enhancement, but no DEF was estimated. Lechtman et al. (2011) (*single and compound*) analyzed GNPs (1.9–100 nm) under brachytherapy and external beam sources using MCNP5 and PENELOPE. MCNP5 modeled photon energy spectra and absorption rates, while PENELOPE simulated electron transport and escaping photoelectric products. Dose enhancement was expressed in terms of GNPs per cell and mass per tumour gram, but without explicit radial DEF calculations. Results aligned with (Jones et al. 2010) and (Leung et al. 2011). McMahon et al. (2011b) (*single*) used Geant4-DNA to simulate dose deposition around a single 2–50 nm GNP in water irradiated by monoenergetic x-rays (20–150 keV) and a 160 kVp spectrum. Auger electrons dominated near the GNP but declined beyond 200 nm, while *L*-shell Auger electrons contributed most. The radial absorbed dose ranged from 3000 Gy at 1 nm to 4 Gy at 300 nm for 20–50 nm GNPs, exceeding 10^5^ Gy for 2 nm GNPs. Cai et al. (2013) (*compound*) simulated photon and electron (nine monoenergetic x-rays: 10–100 keV, a 100 kVp x-ray beam, and ^125^I and ^103^Pd brachytherapy seeds) transport in cell models (single cell, monolayer, and cell cluster) with GNPs of 5, 30, and 50 nm placed in various locations. DEF was calculated for the nucleus (DEF = 3.4, 1.6, 1.7, 2.1) and the cell (DEF = 2.0). The study highlighted larger GNPs in the nucleus maximizing DEF at 15 or 40 keV x-rays. Cell models including cytoplasm, membrane, endoplasmic reticulum, nucleus, and nucleolus with a 400 nm cluster in the cytoplasm and a 300 nm gold layer around the nucleus were investigated using kV and MV photons (Douglass et al. 2012) (*single and cluster*), simulations showed DEFs of 7.0 (cytoplasm, 80 kVp) to 1.3 (cytoplasm, 6 MV), 55.7 (nucleus, 80 kVp) to 2.6 (nucleus, 6 MV), with a DEF of 14.5 in the whole cell for 80 kVp. Lechtman et al. (2013) (*single and compound*) expanded upon prior simulations (Lechtman et al. 2011) to predict sensitizer enhancement ratios using the local effect model. No new DEF values were provided in this study. To study the effect of flattening filter free (FFF) and standard (STD) delivery on endothelial cellular dose enhancement with GNPs, a clinical 6 MV Linac photon beam was simulated (Detappe et al. 2013) (*compound*). The results showed that DEF increased from 1.2 to 4.5. Garnica-Garza (2013) (*single*) calculated microdosimetric spectra, dose-mean lineal energy (*y*_D_), and quality factor (Q) using the PENELOPE code without reporting specific DEF.

Zygmanski et al. (2013) (*single and multiple cluster*) comprehensively reviewed, at that time, the MC simulations on GNP dose enhancement focusing on DEF for single and multiple GNPs in different geometries in macroscopic and microscopic levels using track structure code Geant4 (version 4.9.4) and analytical CEPXS/ONEDANT (Lorence et al. 1989; Lorence and Beutler 1997). They concluded that a 100 nm^3^ GNP irradiated by 100 keV photons from 5 μm showed DEF of 42 in the first 10 nm voxel, decreasing to 2 at 300 nm. Using beams smaller than the GNP overestimated DEF; beams should be at least 10 times the GNP size. For spherical GNPs, DEF was isotropic but energy-dependent, with DEFs of 70 and 10 for 20 and 100 keV beams, respectively. For multiple GNPs, DEF was 20–24 in the first voxel for 50 nm to 118.75 nm separation, decreasing to 1.0 beyond 250 nm. With a 20 keV beam, DEF peaked at 95 for closely packed GNPs.

In the following years, various investigators studied and examined the similar scenarios with single and cluster GNPs under different radiation geometries. Li et al. (2021) (*single and compound*) computed DEF around single GNPs of various diameters (2–200 nm) irradiated by x-rays (60–140 kVp). In the nanometer scale, DEF ranged from 500 at 10 nm to 80 at 200 nm, and in the micrometer scale, from 60 at 1 μm to 1.0 at 20 μm. For tissues with varying gold concentrations, DEF was largely independent of x-ray spectra and gold concentration: at 0.1 w% gold, maximum DEF was 2 at 80 kVp; at 0.4 w%, it was 5 at 60 kVp. Comparing gold foil and GNPs, DEF for gold foil with 60 and 90 keV photons was 40 at 1 μm, while DEF for GNPs irradiated with 60 kVp x-rays was 105, significantly higher than for the foil. Lin et al. (2014) (*single*) simulated GNP-enhanced DEF using the TOPAS MC code for both MV and kV photons and showed DEF from 6 at the GNP surface to 13 at 5000 nm, constant up to 10 mm with 6 MV and 150 kVp beams. For keV photons (50–250 keV), DEF ranged from several hundred at the first nm to 1000 at 100 nm. For 6 MV beams, DEFs ranged from 10 to 20 at 50–100 nm to 300 at 10 mm. Casta et al. (2015) (*single*) compared electron emissions from GNPs and gold planes and found that GNPs emitted 2.3–5.1 times more electrons for a given photon flux. Xie et al. (2015) (*single and compound*) showed x-ray enhanced DEFs for single GNPs ranged from 1000 to 10,000 near the GNP surface, dropping to 10–20 at 100 nm. For multiple GNPs, the highest DEFs were over 10 near the nucleus, and around 5 in the cytoplasm. Randomly distributed GNPs in the cytoplasm had DEFs mostly between 1 and 2, with an average of 1.1. These spikes were similar to the nucleus DEF of 3.5 simulated by (Cai et al. 2013). Kirkby and Ghasroddashti (2015) investigated the GNP-induced dose enhancement effects in mitochondria-targeted radiotherapy for cancer cells using MC simulations without reporting DEF.

Incerti et al. (2016) provided an atomic deexcitation package to simulate full Auger cascades in the Geant4 code. They tested the package for 10–100 nm GNPs with photon energies of 10 and 100 keV, observing significant improvements in low-energy electron spectra associated with gold’s outer shell Auger transitions, with less than 1% change in emitted energy.

The research group around Kirkby investigated the enhancement effects of GPNs with different forms in the radiation therapy. Koger and Kirkby (2016b) (*compound*) investigated photon beam energy effects on GNP-enhanced arc radiation therapy (GEART) using the PENELOPE MC code and showed that GEART treatments achieved up to 88% higher mean tumour doses with a 50 keV source compared to a 6 MV treatment. Kirkby et al. (2017) (*single and cluster*) simulated energy absorption from a single GNP irradiated by monoenergetic photons, focusing on local and nonlocal energy deposition within 500 nm and beyond in water surrounding a GNP cluster. They found reduced energy deposition in tightly packed clusters, especially at low photon energies (< 40 keV). In a hexagonal close-packed (HCP) cluster with 90 additional GNPs, local deposition decreased to 29%, and nonlocal deposition reduced to 26%. Koger and Kirkby (2017) (*cluster*) simulated the dosimetric effects of polyethylene glycol (PEG) surface coatings on GNPs. In microscopic simulations, they found that PEG coatings up to 20 nm thick reduced dose deposition by 2–7.5%, depending on GNP size. Nanoscopic simulations showed dose reduction within 500 nm of a single GNP by 5–34%, depending on size, energy, and coating thickness, thus highlighting the impact of coatings on dose enhancement.

Ferrero et al. (2017) (*compound*) and Strigari et al. (2017) (*compound*) used a 2 nm GNP placed at the center of a 10 × 10 × 10 mm³ water phantom irradiated by photon beams (160 kVp, 6 MV, and 15 MV). The energy deposition and dose were calculated within a 1 nm radial bin around the GNP surface for RBE, cell survival, and TCP modelling, however no DEF was reported. Sung and Schuemann (2018) (*single and multiple*) explored the enhancement effects of kilovoltage (50–250 kVp) and megavoltage (6 MV) photons on GNPs using the TOPAS-nBio MC code. They modeled cell survival for brain and breast tumours based on radial doses and a GNP-local effect model. For brain tumours, GNPs were most effective above 50 keV with multiple beams. For breast tumours, a single-kV beam increased RBE-weighted dose by up to 2.7 times compared to megavoltage beams, suggesting that small, shallow tumours benefit most from GNP-enhanced radiation therapy. Kwon et al. (2018) investigated energy absorption by clustered gold nanoparticles and their results suggest that previous simulations assuming a homogeneous GNP distribution have overestimated the dose enhancement, especially for smaller GNPs and higher concentrations. Vlastou et al. (2020) (*single and multiples*) reviewed the dose enhancement with various GNPs and MV photons from 2010 to 2019. They showed that GNP concentration affects DEF, but GNP size alone has an unclear effect. GNP location and distribution within tumours are crucial for accurate MC simulations. Clustering distribution of GNPs, rather than homogeneous models, should be prioritized for more realistic in vivo simulations. The kV photons were found to achieve greater dose enhancement than MV photons. FFF photon beams were also recommended for more effective GNP dose enhancement. In a simulation work on *single and multiple* GNPs, Engels et al. (2020) showed DEFs of 22 and 48 for 15 nm and 100 nm GNPs, respectively, within 1 nm irradiated by 150 kVp x-ray beam (10 μm lateral size). Including Auger electrons in simulations increased the DEF for the 100 nm GNP by 50% up to 500 nm.

In a framework of EURADOS computational exercise, Li et al. (Li et al. 2020a, b, 2021) (*single*) simulated a single GNP (50 nm and 100 nm) irradiated by x-rays (50 kV and 100 kV) in liquid water using various MC radiation transport codes. The median DEFs in 10 nm-thick water shells ranged from 400 to 600 for 10 nm GNPs, decreasing to 40–100 for larger GNPs and varying x-ray spectra. Rabus et al. (Rabus et al. 2019, 2021) (*single*) showed that radiation transport simulations in (Li et al. 2020a, b) using narrow-beam geometry overestimate absorbed dose and DEF due to not accounting for second electron equilibrium (SEE). For spherical GNPs (50–100 nm) and 50–100 kVp x-rays, maximum DEFs were estimated to be 10 and 20, lower than the values from the EURADOS exercise. Dose enhancement was confined to 100–200 nm around the GNP, with scattered photons having little impact.

In their comprehensive book, Sajo and Zygmanski (2020a) provide a thorough overview of nanoparticle-enhanced radiation therapy. In Chap. 1 (Sajo and Zygmanski 2020b) (*single and multiple*), they explain the Auger process and the role of fluorescent photons in dose enhancement. When photons interact with GNPs, fluorescent photons are emitted, which can lead to further photoelectric effects and Auger processes, enhancing the dose both within and outside the GNPs. This photoelectron cascade is crucial for dose amplification, comparable to the effect of Auger electrons. In Chap. 2 Sajo et al. (2020a) (*single*) studied the DEF for single spherical GNPs (10, 30, 60, 100 nm) irradiated by various monoenergetic and spectral photons at depths of 2 and 20 cm in a water phantom. They found that GNP depth in water had minimal impact on DEF, and DEF values were calculated for photon energies from 14 keV to 6.5 MeV. The maximum DEFs for 10, 30, and 100 nm GNPs near the surface were 56, 89, and 119 for monoenergetic photons, respectively. For 120 and 220 kVp x-rays, DEFs ranged from 22 to 65 for the three sizes. For 6.5 MV photons, DEFs ranged from 1.8 to 3. In Chap. 3 Sajo et al. (2020b) (*multiple*) studied the DEF at a distal centimeter from multiple GNPs in various densities was evaluated using 20–400 keV photon beams. For a 100 keV photon beam, the maximum DEFs for GNP concentrations of 40, 90, 300, and 830 Au mg g^− 1^ were 5, 9, 28, and 75, respectively. In the cluster model, GNPs formed groups with densities of 640 Au mg g^− 1^, resulting in DEF peaks of 7, 1.6, 2.4, and 1.06 for photon energies of 20, 80, 120, and 400 keV, respectively.

Poignant et al. (2021) (*single*) introduced the probability enhancement ratio (PER) to estimate the likelihood of imparting specific energy exceeding thresholds (1, 10, and 20 kGy) to nanometer-scale targets, normalized to pure water. This was calculated using the MC code MDM, with the biophysical code NanOx (Cunha et al. 2017) determining threshold values. The PER was influenced by factors such as GNP radius, photon energy, threshold *z*_0_, and distance from the GNP surface. The highest PER of 95 occurred with a GNP radius of 50 nm, 20 keV photon energy, 20 kGy threshold, and a target 5 nm from the GNP. However, PER decreased as the distance from the GNP increased, highlighting the importance of precise GNP placement for optimal therapeutic outcomes.

During 2021–2023, many simulation investigations with GNPs in various forms were reported. Using the MC code EGSnrc and the egs_brachy user code (Kawrakow 2000; Chamberland et al. 2016), Simiele et al. (2021) (*single and compound*) obtained DEFs of 1.06 and 1.00 for IC 80 and 150 keV beams, respectively. Kolyvanova et al. (2021) (*compound*) achieved a DEF of 2.51 for GNPs irradiated by 237 x-ray spectra at 30–300 kVp with various filtrations. Melo-Bernal et al. (2021) (*single*) reported a DEF for GNPs in MDA-MB-231 cells irradiated with 40 keV x-rays, which is comparable to those of McMahon et al. (2011a) applying a two-step method combining MC simulations and an analytical approach. Kim et al. (2021) (*multiple*) developed a microdosimetric kinetic model (MKM) for GNP radio-enhancement (GNP-MKM), incorporating quantities like dose-mean lineal energy, estimated using the MC code Geant4 and computed a sensitizer enhancement ratio (SER) of 1.41 for a 500 nm domain radius. Jabeen and Chow (2021) simulated scenario of a single GNP (diameters of 30, 50, and 100 nm) positioned near DNA irradiated by photon beams (50, 100, and 150 keV) at various distances in the presence of a magnetic field found DEF values ranged from 4.5 to 7.5 without a magnetic field and slightly increased DEF with a magnetic field (0–2 T). Daems et al. (2021) carried out a review about radiolabeled GNPs and their general application in nuclear medicine imaging and image-guided therapy, especially the enhancement of targeted radionuclide therapy and nano-brachytherapy. The review presented a summary of DEF (from 1.00 to 271.48) in tumour using the MC simulation for GNPs, in different concentrations and sizes, combined with different radionuclides, such as ^125^I, ^192^Ir, ^169^Yb, ^103^Pd, ^60^Co, ^198^Au and ^137^Cs. Most DEF values are in the range of 1.00 to 2.00. The greatest values of DEF are 271.48, 219.91, and 99.6 where 400 nm diameter GNP in a relatively high concentration (350 mg g^− 1^) irradiated by ^103^Pd, ^125^I and ^169^Yb, respectively (see Supplementary Table 1). Robatjazi et al. (2021) simulated the interactions of eight different 50 nm nanoparticles (Ag, Au, Bi, Fe, Gd, Hf, Pt, and Ti) when irradiated by mono-energetic photon beams of 30, 60, and 100 keV. They obtained the maximum DEF values for GNPs of approximately 1000, 600, and 2000 at 30 keV, 60 keV, and 100 keV photon energies, respectively. For 60 keV photons, the DEF reached up to 3000 at a distance of 3000 nm from the GNP surface.

In the following, the simulations mainly focused on evaluation of enhancement effect of GNPs in compound and multiple forms in the clinical radiotherapy scenarios. Schmidt et al. (2022) (*single and multiple*) carried out a study on the effect of PSMA-targeted GNP for treating prostate cancer with 6 MV beams using the TOPAS/Geant4 package and found DEF of 1.14 and 1.55 at different depth. Spina and Chow (2022) (*compound*) investigated the dose impact of flattening filters (FF), with GNP concentrations ranging from 3 to 40 mg ml^− 1^ in radiotherapy, using 6 and 10 MV FF- and FFF-photon beams from a Varian TrueBEAM linear accelerator. The results showed that the maximum DEF for 6 MV beams was 1.08 with FF and 1.14 without FF. For 10 MV beams, the maximum DEF was 1.04 with FF and 1.07 without FF.

Xu et al. (2022) (*single and compound*) studied the DEF of two metal nanoparticles, one core-shell magnetic GNP, Fe_3_O_4_@AuNPs and GNPs, in single NP (100 nm diameter) and concentration forms in a spherical cellular model, irradiated by x-rays photons (50, 100, 150, 200, and 250 keV) using the MC code TOPAS-nBio. The simulations showed that DEF of Fe_3_O_4_@AuNPs was lower than that of GNPs in a single NP and as well in a cell irradiated by monoenergetic photons. However, the results demonstrated that in the cellular geometry under magnetic field, DEF of Fe_3_O_4_@AuNPs irradiated by a clinical brachytherapy source was 22.17% for the cytoplasm and 6.89% for the nucleus higher than those of AuNPs at the concentration of 50 mg ml^− 1^. Sadiq and Chow (2022) (*compound*) examined the impact of bone scatter effects on metal nanoparticle (MNP) dose enhancement in skin lesions utilizing 105 kVp and 220 kVp x-rays from a Gulmay D3550 unit and the EGSnrc MC code. The DEF of 5.9 was observed for GNPs at 40 mg ml^− 1^ concentration irradiated by 105 kVp x-rays. Mesbahi et al. (2022) (*multiple and compound*) calculated the DEFs for several MNPs, including GNPs (20 nm in diameter and the concentrations ranged from 1 to 5% w/w) irradiated by Xoft Axxent electronic brachytherapy source (peak energy 50 kVp) in resection cavities of breast tumours using the Geant4 MC code (version v10.07.p02). The DEF values for GNP are found to be 2.3 to 5.9, and the DEF values are increased almost linearly with GNP concentrations. López-Valverde et al. (2022) (*compound*) applied the EGSnrc code for the simulation of one single 15 nm diameter GNP irradiated by two different beams (6 MV and MV-CBCT photon beam) generated from a Siemens ONCOR™ accelerator for enhanced x-ray tomography and dose enhancement in treatment. They found DEFs of 1.009 for 6 MV and 1.159 for MV-CBCT. They also concluded that a GNP concentration around 20 mg Au ml^− 1^ is an efficient amount used for diagnosis, treatment planning, and monitoring treatment. Vlastou et al. (2022) investigated the interaction of 100 nm GNP clusters in a water phantom irradiated by 6 MV external photon beams, both with and without a flattening filter, using the GATE code. The study examined the dependence of DEF on GNP size, concentration, and distribution pattern, with DEF values ranging from 0.99 to 1.26. Tegaw et al. (2022) assessed DEFs for GNP-enriched breast tissue using the INTRABEAM^®^ system with the EGSnrc/Epp MC code. They simulated a spherical applicator (1.5 and 5 cm in diameter) made of biocompatible polyetherimide filled with air, inserted into breast tissue. They attained DEFs ranged from 1.00 to 3.6 for a GNP concentration of 20 mg Au g^− 1^ in tissues.

Lin et al. (2023) (*multiple*) studied the dose enhancement effect of x-rays on ^127^I, radio-iodinated deoxyuridine (IUdR), and GNPs using the MCNP6 code. They obtained DEFs of 1.23 and 10.57 for ^60^Co gamma rays and 80 kVp x-rays, respectively. Chow and Jubran (2023) (*compound*) conducted a study on the dependence of the DEF, for several metal nanoparticles, as a function of the depth in a phantom irradiated by 105 kVp and 220 kVp orthovoltage beams and found that the DEF decreases with depth, with this decrease steeper for higher nanoparticle concentrations. Thus, they attributed this behavior to the increment of self-absorption of the solution as the nanoparticle concentration increases. Similar to the work by Chow and Jubran (2023), Martinov et al. (Martinov et al. 2023a, b) (*compound*) studied the dose-enhancement effect of GNP in a microdosimetric environment irradiated by low energy x-rays. They carried out simulations for several nucleus to cytoplasm size combinations and GNP concentrations and implemented a heterogeneous multiscale model for considering detailed models of cell populations. They observed a decrease of the DEF as the GNP increases, due to self-attenuation of the primary photons. Dheyab et al. (2023) (*single*,* multiple and compound*) reviewed the use of MC simulations for studying the dose enhancement effect of GNPs when irradiated with x-rays and gave a further overview on the multi-scale simulations involving the induction of DNA damage, including the indirect damage mediated by chemical species produced by the radiation.

Velten and Tomé (2023) (*single*) simulated the dose enhancement effect produced by 50 nm GNP irradiated by 250 kVp x-rays and spread-out Bragg peak protons combining the PENELOPE MC code for macroscopic dose deposition calculations and Geant4 code for track structure simulations. They applied the local effect radiobiological model and found that survival curves and DEF are in good agreement with the reference data. However, Rabus (2024) evaluated the work (Velten and Tomé 2023) using a local effect model (LEM) approach and argued that the reported DEF values for 6 MV and 18 MV photon irradiation substantially overestimated the number of radiation-induced lesions. Gray et al. (2023) (*single and multiple*) conducted simulations of a single gold nanosphere and a gold nanocube, both of equal volume, irradiated by 6 MV and 18 MV therapeutic photons using the MCNP6 code. The study revealed that the dose from electrons around the gold nanocube exceeded that of the nanosphere by up to 18.5% at 18 MV and 23.1% at 6 MV, within a distance of 1.1 mm. DEFs were found to range from 2 to 8, with the highest DEF observed for the gold nanocube irradiated at 6 MV. However, Rabus et al. (2024) highlighted several issues in the previous study and emphasized that both the MC simulations and the experiments should employ the same GNP concentration for a meaningful comparison. It was also pointed out that Gray et al. (2023) reported a physically unrealistic dose per photon for the irradiation of a 1-µm water cube, as the minimum energy imparted by a single photon interaction is on the order of approximately 10 eV.

Radioactive nanoparticles have also attracted considerable interest for nano-brachytherapy applications. Angelocci et al. (2024) estimated dose rates from radioactive ^198^Au nanoparticles for prostate cancer nano-brachytherapy using MCNP6.2 with both mathematical and ICRP mesh anatomical models. Their results showed that 98% of the tumour dose originated from emitted electrons, demonstrating the feasibility of using ^198^Au nanoparticles to achieve highly localized tumour irradiation while minimizing dose to surrounding healthy tissues.

Comparisons between different radiation modalities have further highlighted important limitations of nanoparticle-mediated enhancement. Azarkin et al. (2024) investigated GNP-enhanced proton therapy and x-ray therapy using Geant4 simulations for nanoparticles ranging from 10 to 80 nm in diameter. They found substantial dose enhancement near GNPs for x-ray therapy, whereas enhancement in proton therapy remained modest. Moreover, realistic therapeutic GNP concentrations produced relatively small macroscopic dose enhancement factors across all treatment scenarios.

The relative contribution of photons and electrons to nanoparticle dose enhancement was investigated again by Kim et al. (2024), using MCNP6.2 and Geant4 simulations for Auger-emitting radioisotope-labeled nanoparticles. Their results showed that the maximum photon DEF occurred at 20 keV, reaching values of 63.4 using MCNP6.2 and 50.5 using Geant4. However, the physical dose enhancement expected from photon interactions was substantially reduced because electron contributions dominated radial dose deposition near the nanoparticle surface.

At the microscopic level, Thomas et al. (2024) explored ionization clustering around gold nanoparticles using MC track-structure simulations. They demonstrated that low-energy electrons emitted from nanoparticles significantly increased local ionization clustering beyond what would be expected from energy deposition alone. Their findings suggested that nanoparticle dosimetric effects are governed by the interplay between secondary electron production and the ability of these electrons to escape the nanoparticle.

Rasouli and Masoudi (2024) used the MCNPX code to investigate GNP accumulation in healthy tissues of a human eye phantom. They observed DEF values as high as 6 in superficial layers, decreasing to approximately 1.2 in deeper layers. Significant dose deviations were also found in critical ocular structures such as the iris, cornea, sclera, and lens.

Biological mechanisms beyond purely physical dose enhancement have also been increasingly recognized. Hernández Millares et al. (2024) investigated iron oxide and gold nanoparticle-induced radiosensitization in A549 lung cancer cells irradiated with 6 MV and 300 kVp x-rays. Their findings demonstrated increased cell killing for both nanoparticle types under both irradiation conditions and suggested that radiosensitization mechanisms resemble ferroptosis induction rather than relying exclusively on physical dose enhancement.

Nanoparticle geometry also plays an important role in secondary electron emission. Taheri et al. (2024) assessed gold nanorods using the TOPAS track-structure code and showed that gold nanorods exhibit secondary electron emission comparable to spherical nanoparticles. However, nanoparticle geometry significantly influenced the emission of low-energy M-shell Auger electrons below 3.5 keV, suggesting potential applications in combined photothermal therapy and radiotherapy.

Realistic cell geometries have further improved the understanding of nanoparticle effects at the cellular scale. (Antunes et al. 2024) constructed voxelized glioblastoma cell geometries from confocal microscopy images and evaluated both DEF and sensitization enhancement ratio (SER) using MC simulations and a modified linear-quadratic model. Their results demonstrated that the intracellular gold volume fraction is the dominant parameter governing both DEF and SER, with nanoparticle size having a greater impact than the absolute number of nanoparticles.

Ghalamchi et al. (2025) reviewed DEFs for various NPs in MV radiotherapy using both in vitro experiments and MC simulations. Their analysis showed that experimentally measured DEF values were generally higher than those predicted by MC simulations. Among the investigated systems, GNPs with a diameter of 100 nm and a concentration of 6 mM irradiated by 6 MeV photon beams exhibited the highest DEF values in both experimental and simulation studies, with a maximum reported DEF of 4.71, corresponding to a 53% dose increase in a 6 mM gel system. Similarly, Movahedizade and Sajjadi (2025) investigated the dose enhancement potential of Au, Pt, and Bi nanoparticles at concentrations of 3 and 7 mg g^− 1^ using multi-scale simulations with the Geant4 and Geant4-DNA toolkits. Their results demonstrated consistent DEFs of up to 2.6 at both macroscopic and microscopic scales for NP including gold.

The influence of beam energy and self-absorption effects was investigated by Alba et al. (2025), who used the PENELOPE code to study gold, silver, platinum, and bismuth nanoparticles irradiated with 100 kVp and 6 MV photon beams, as well as gamma rays from ^192^Ir and ^60^Co sources. Their simulations showed that DEF values are maximized for low-energy photon beams, high atomic number materials, high NP concentrations, and small nanoparticle sizes with self-absorption effects and energy loss within the nanoparticle itself.

Several studies combined experimental radiobiology with advanced MC modeling. Teixeira et al. (2025) experimentally demonstrated that GRPR-targeted gold nanoparticles carrying bombesin peptides (AuNP-BBN) represent promising nanoplatforms for glioblastoma (GBM) therapy. Accompanying MC-based radiobiological simulations using realistic microscopy-derived glioblastoma cell models were consistent with the experimental findings and further indicated that cells were more sensitive to x-rays than to gamma radiation. Parwaie et al. (2025) evaluated dose enhancement ratios at both macroscopic and microscopic scales for different radiation qualities and GNP concentrations using the OncoSeed ^125^I model 6711 spectra and simulated Linac MV beams. The results demonstrated that under 50 keV photon irradiation, increasing GNP concentration from 1 to 30 mg g^− 1^ produced dose increases of up to 2.29 for photons and 1.22 for electrons. Increasing beam energy significantly reduced DEF values, whereas increasing GNP concentration substantially enhanced the effect.

The influence of nanoparticle coatings has further emerged as an important topic. Taheri et al. (2025) investigated the effect of coating layers on the radioenhancement efficiency of gold nanorods using the TOPAS code with 100 kVp x-rays and showed that coating layers reduced the additional dose generated by gold nanorods by up to 7%, suggesting that coating effects on physical radioenhancement may be less pronounced than previously assumed. Consistent findings were reported by Mansouri et al. (2024), who simulated polyethylene glycol-coated bismuth, gold, iridium, and gadolinium nanoparticles for photon beams of 30, 60, and 100 keV using Geant4. They found that thicker coatings reduced secondary electron emission, decreased DEF values, and also influenced water radiolysis and reactive species production.

Araujo and Fonseca (2025) used a high-resolution voxelized in vitro culture medium model with the MCNP6.2 code to simulate a high-dose-rate (HDR) ^192^Ir brachytherapy scenario. Their results demonstrated a proportional relationship between DEF and nanoparticle concentration, enabling the derivation of a predictive equation for DEF estimation that agreed well with published data. At the nanoscale, Kaphle et al. (2025) investigated radiation-induced DNA damage enhancement from intracellular GNPs using a nanoscale cellular MC model under both 250 kVp and 6 MV photon irradiation. They observed increased nucleus dose deposition and enhanced DNA damage in the presence of GNPs, with maximum dose enhancements of 4.24% and 4.34% for 250 kVp irradiation, and 3.04% and 3.22% for 6 MV irradiation. Indirect SSB and DSB yields were significantly higher than direct damage yields, emphasizing the dominant role of indirect radiation damage mechanisms. The 250 kVp beam produced greater SSB enhancement, whereas the 6 MV beam yielded stronger DSB enhancement.

Self-absorption by the GNP is an important question in this context as aforementioned. Alba et al. (2025) studied the impact of this phenomenon on the dose enhancement around metallic nanoparticles when irradiated by kVp x-rays, 6 MV, ^192^Ir, and ^60^Co photons. They used the PENELOPE MC code and found that the DEF decreased by 9.5% when the GNP size increased from 2 nm to 100 nm, during a 100 kVp x-ray irradiation, keeping the gold concentration constant. For high energy photons, 6 MV and ^60^Co, this decrease was only 0.1%. It would be expected that the higher the incident photon energy, the higher the energy of secondary electrons, which would be less absorbed by the GNP. A study reported by Jayarathna et al. (2024) focused on the emission of secondary electrons from GNP irradiated by 6 MV photons. They developed a nanometric-resolution cell model using transmission electron microscopy, starting from a real cell image, containing a certain GNP distribution. In addition, they created a formalism based on nanometric point dose kernels in order to determine dose distribution inside the cell due to secondary electrons. Each GNP was regarded as a secondary electron source. This approach allowed the determination of the dose distribution inside the cell due to the photon irradiation, in the presence of GNPs. No DEF was determined in this work.

#### Radionuclides and brachytherapy

Some radionuclides have been investigated for their utility in the so-called targeted radionuclide therapy. In this treatment modality, a beta or alpha emitter is conjugated with some vector or carrier that is preferentially accumulated in the target tumour. One of the main problems faced when using alpha emitters, is that the daughter recoil energy is much higher than the binding energy with the vector, so that the radionuclide detaches from the carrier just after the decay, which is an undesirable effect in this treatment modality when there are more radioactive daughters in the decay series. Depending on the daughter isotope half-life, it could decay far away from the target. McLaughlin et al. (2013) proposed a solution for this problem, coating the radiolabeled conjugate with a thin gold shell, conforming a complex GNP. More works on radionuclide and nanoparticle conjugation can be found in the review by Trujillo-Nolasco et al. (2021). Gholami et al. (2019) showed that conjugating radionuclides with some metallic nanoparticles can produce a dose enhancement up to 20% around the particles. However, GNP was not used in this study, which could be a research topic in future.

Silva et al. (2021) studied, in vitro, the radiobiological enhancement of GNP radiolabeled with ^177^Lu. They used the MCNP MC code and two irradiation setups: (1) a single GNP in the center of a spherical cell nucleus with a uniform activity distribution inside; (2) a thin nanometric gold spherical shell just at the nucleus-cytoplasm frontier, with the activity uniformly distributed in the cytoplasm. They found that the DEF due to x-ray emission from the GNP goes from about 6 down to 2, when the distance from the GNP surface varies from 10 to 60 nm. However, DEF due to Auger, beta, and internal conversion electrons remains around 1.5 along the same range.

Farahani et al. (2019) focused first on superficial tumours and later studied deep-lying tumours, where higher energies are required due to their greater penetration power. The interest of the study is the dosimetry measurement they provided using MAGAT polymer gel. Experimental dose enhancement was measured in polymer gel with and without GNPs. The dosimeters were irradiated using two different sources, the ^192^Ir is mainly a gamma emitter (380 keV) and the ^60^Co emitting 1.25 MeV photons. Their results showed an increase in the polymer modification, the enhancement caused by GNPs becoming more important for higher doses. Both sources of irradiation showed the same enhancement tendency. It is worth mentioning that the concentration of GNPs was 0.2 mM which tolerability in biological cells is to be verified, however since their experiment was conducted on gel dosimeters the toxicity issue was not mentioned.

#### Synchrotrons

Synchrotron radiation offers a unique opportunity to investigate x-ray interactions with GNP because it provides highly intense, quasi-monochromatic photon beams with finely tuneable energies. This allows irradiation at specific atomic absorption edges, particularly the gold K-edge, enabling the contribution of photoelectric absorption and associated secondary electron production to be quantified. In this way, the DEF can be systematically evaluated as a function of photon energy. However, DEF is not solely determined by beam energy; it is a multidimensional quantity that also depends on nanoparticle characteristics, spatial distribution, biological localization and irradiation geometry considered. Rahman et al. (2012) carried out a work to determine the DEF for GNP using a normoxic type polyacrylamide (nPAG) gel dosimeter. This dosimeter correlates the absorbed dose with the polymerization degree of the gel in question. They doped such a gel with GNP and irradiated the medium with a synchrotron radiation beam of 125 keV and 80 kVp x-rays from a superficial therapy machine. They found experimental DEF of about 1.76 and 1.64 for the synchrotron radiation and therapy beams, respectively. Uncertainties were of the order of 20–25%. They also irradiated the gel dosimeter with 6 MeV electrons and 6 MV photon beams and obtained DEF of 1.37 and 1.14, respectively.

Gagliardi et al. (2020) also studied the absorption of synchrotron radiation from GNP. They used the radiochromic polyurethane dosimeter PRESAGE to determine DEF experimentally. They loaded the dosimeter with GNP in diameters of 5 and 50 nm and irradiated the medium with beams of 95.3 keV mean energy synchrotron radiation, 150 kVp superficial x-rays, and 6 MV x-rays, for which they found DEF in the ranges 1.16–1.32, 1.12–1.21, and 1.02–1.05, respectively.

### Electrons

Studies of electron beam effects on nanoparticles and their implications on biological media are still rare. This might be due to the wider application range using photon irradiations and it shows a higher interest for photon and heavy ion irradiations among the radiation biology community. To our knowledge, one of the first investigations on GNP irradiations started with Zheng et al. (2008) where thin films of plasmid DNA were irradiated with 60 keV electrons with and without GNP in the irradiation medium. Although the title describes high-energy irradiations, it is important to note that this depends on the field of studies, 60 keV electrons are considered low energy particles when working in radiation therapy, however, they might be considered of high energy in radiobiology studies. Moreover, in radiobiology the main interest concerns subcellular structures and effects of radiation on particular molecules, such as the DNA, and therefore particles’ energies remain important even for values as low as 100 eV for electrons. In their study, Zheng et al. focused on single and double strand break analysis after irradiation, and measurements were carried out using the gel electrophoresis technique. The high-resolution analysis of nanoparticles revealed a size distribution of 1 to 12 nm with 87% of the particles smaller than 5 nm. It was also demonstrated that the size of GNPs varies with time, since these particles tend to aggregate after a few days of storage; therefore, their solution was prepared and mixed within 12 h from the experiment to preserve the estimated particle size distribution. In addition, the average diameter of 3.7 nm changes when DNA is present in the solution because DNA and GNPs are attracted to each other, which modifies the GNPs aggregation properties. The results were compared between irradiations differing in electron numbers and GNP versus plasmid ratios. The number of DNA strand breaks increased systematically with increasing number of incident electrons and increasing GNP concentration. The most interesting result is that a small amount of GNP is required to enhance DNA damage, in fact, one GNP per 30 DNA molecules is sufficient to cause an increase of 10% in SSB numbers and 3% increase in DSB numbers per DNA molecule. On the other hand, it was also shown that even non-irradiated DNA showed an increase of strand breaks numbers in GNP solution, which also confirms the toxicity of GNPs, probably enhancing the chemical sensitivity of the DNA. In this study, GNPs were directly bonded to the DNA molecule; therefore, the generated low energy electrons had high probability of reaching parts of the DNA and inducing damage. This is particularly important in comparison with other experiments of cellular irradiations where GNPs might be prevented from entering the cell nucleus depending on their size. In cellular experiments, localization of GNPs can be considered different from in DNA experiments, and the average range of produced electrons might not be sufficient to reach the DNA molecule in some cases. At the same time, for irradiations where GNPs are bound to DNA, high-energy electrons might not induce further DNA damage. Due to their large penetration depth, most of their energy is deposited far from the DNA. In the experiment, Zheng et al. (2008) consider that most of the damage is caused by 8 to 68 eV electrons which range from 0.8 to 2.5 nm.

Another study published by Chang et al. (2008) investigated the potential biological effects of GNPs for electron single-dose irradiations on tumours in mice. The study was targeting clinical applications for melanoma cases. GNPs of spherical shapes and 13 nm size were used, and the concentration was approximately 180 Au µg ml^− 1^. The tumour irradiations with 6 MeV electrons, took place 24 h after NP solution injection. The results showed an increased biological effect of radiations in groups injected with GNP solutions. Tumours were excised from mice 6 h after irradiation and the apoptotic activity was measured taking into consideration four groups of tumours; control, GNP injected but without irradiation, irradiated tumours without GNP injection and tumours combining GNP injection and irradiation. The results showed that the number of apoptotic cells increased by around 75% when GNP particles were combined with irradiation.

Since the range of secondary particles plays an important role along with nanoparticles distribution, brachytherapy applications were also investigated. In this type of therapies, the source is located within the tumour or very close to the tumour location therefore delivering high local doses. MC simulations were carried out by Zhang et al. (2009) using the Geant4 MC simulation toolkit (Agostinelli et al. 2003; Allison et al. 2006) version 4.8.1. An Ir-192 source is placed in a water phantom and 100 nm spherical GNPs were added with a concentration of 10^13^ cm^− 3^ and the dose enhancement effect was calculated. The source decay process generates electrons with a probability of 95.1% along with photons generated from electronic shells deexcitation. The study reported a radial dose enhancement around the source varying between ~ 20% to ~ 35% with the radius. The study also compares two scenarios, the first where GNPs are of spherical shape and uniformly distributed in the medium and the second using a homogeneous water-gold medium taking into account the mass ratios for the mixture. The latter case was shown to overestimate the dose enhancement. Therefore, in conclusion using approximate models of elements, mixture is not suitable for this type of calculations. The dose enhancement overestimation starts to be significant above 10 mm radius and reaches a maximum of 15% around 17 mm from the source.

Chow et al. (2012) investigated secondary electron production from GNPs irradiated with electron beams. The incident beam was considered monoenergetic and the study included beams of energies equal to 50 keV, 250 keV, 1 MeV and 4 MeV. Therefore, these energies range from radiation biology applications up to radiation therapy energy values. Also, the spherical GNP size included diameters of 2, 50 and 100 nm. They used the MC simulation toolkit Geant4 to detail the electron tracks directions and the energy depositions inside and around the GNP volumes. They have shown that the effective range of electrons, produced in the GNP and traversing out to the water medium, increased with the energy of the incident beam. This is somehow expected since the maximum energy transfer, between the incident electron and the target, increases with the incident particle’s velocity. In addition, they have shown that the effective range increases with the diameter of the nanoparticle, and this is more pronounced for higher incident energies. Therefore, for a 4 MeV electron beam the range increased from less than 1 μm when using 2 nm diameter GNPs up to ~ 15 μm when using 100 nm GNPs. This also revealed a higher energy deposition in the irradiated medium; the enhancement increased with the nanoparticle diameter and with the incident beam energy as well. The total secondary electron energy deposition was higher for 50 keV irradiations, because low energy electrons have higher interaction cross sections, and this was reflected in the results while the normalised values per unit mass and per electron flux showed higher energy depositions for 4 and 1 MeV energies and a decreasing deposition with increasing GNP diameter. However, this study didn’t show total dose enhancement results but focused more on the properties of the electrons around the nanoparticles which was commented on by Rogers (2013) stating that it is irrelevant whether the dose enhancement is really occurring from the secondary particles and this puts a doubt on the advantage of using GNPs for electron beam irradiations. Moreover, Rogers showed that statistical uncertainties on the dose from nanoparticle generated secondaries is greater than 25% taking into account that the probability of an electron hitting the GNP of 100 nm within a cubic volume of 1.5 mm is less than 1 per 10^6^. Therefore, the probability that the dose enhancement effect will be observed in realistic conditions is even lower because irradiated volumes are usually cellular tissues or organs and have a wide size. Mainly the dose delivered by the secondary electrons generated from the nanoparticles is negligible in comparison with the total dose in the medium. This is not the case for photons where the interaction cross section is roughly 600 times higher for gold than that for water, hence the interesting observed effects in photon irradiation experiments. This is not the case for electrons, these are charged particles, and they interact continuously in any medium, therefore the presence of GNP does not bring any addition to the energy deposition. In reply to the comment of Rogers (2013), Chow et al. (2012) justified the fact that the dose enhancement due to the presence of GNPs was not calculated because indeed it is not significant. Their research was focused on showing the properties of secondary electrons generated around GNPs, and even if the dose enhancement is negligible, the obtained results can still be of interest for radiation biology where the chemical properties of the irradiated medium are of importance, and secondary electrons in this case can play an important role by enhancing free radical production.

In an effort to make nanoparticle usage more accessible in radiation therapy, Koger and Kirkby (2016a) established a calculation model that can predict DEF without explicitly simulating individual GNPs. In their work, they used 2 simulation methods, the first one considering a homogeneous gold and water mixture and the second detailing explicitly each GNP volume in the medium, this would allow later an easy conversion between the two methods in case GNP-infused geometry is used due to limited computational power. In fact, modelling a number of GNPs generates a high number of volume boundaries to be considered in the simulation which can require high computational resources. Calculations were carried out for photon and electron beams and energies ranging between 150 eV and 2 MeV. Spectra of the beam energies were obtained using dedicated software and the source energy distributions were considered in the simulations. Calculations were carried out using the PENELOPE MC software (Salvat et al. 2012) and considering different GNP diameters; 2, 10, 20, 50 and 100 nm and different GNP concentrations; 5, 10, 15, 20 and 30 mg Au g^− 1^ tissue. The tissue dose ratio between using explicit GNP volumes and considering a homogeneous mixture of GNP and tissue was calculated for all the mentioned parameters. Calculated DEFs depended on the energy of the incident beam and on the size of the nanoparticles, the values ranged from 0.69 to 1.04 for photons and from 0.97 to 1.03 for electrons. The main advantage of this work is that it allows complex simulations of dose depositions without the need to model each GNP in the medium and this reduces the dependency on computational power.

The dose enhancement effects of nanoparticles are only of interest if relevant for medical applications, as an attempt to quantify the radiosensitizing effect of nanoparticles (Mousavi et al. 2017) measured the radiosensitivity enhancement of melanoma cells irradiated with megavoltage electrons. The results showed a decrease in cell survival ratios with increasing GNP concentration. The size of the used GNPs was around 50 nm and the cellular uptake occurred at all concentrations. In fact, two concentrations were used; 3 and 30 µM and the melanoma cultures were irradiated at different doses; 2, 4, 6 and 8 Gy with 6 MeV electrons. The use of an electron beam in the case of melanoma is advantageous because skin cancer is relatively superficial, and a surface energy deposition is needed. Sphericity and size of synthetized GNPs were verified using transmission electron microscopy and dynamic light scattering respectively. Survival curves showed an increased sensitivity for cells with GNPs and the surviving fraction decreased with increasing GNP concentration. Considering the previously mentioned studies showing that the dose enhancement in the case of electrons irradiations was minimal suggested that the observed increase in sensitivity was rather due to either chemical or biological effect as also mentioned by Mousavi et al. in their conclusion.

Also Mehrnia et al. (2017) conducted an experimental study using GNPs in 4 MeV electron beam irradiation for breast cancer cells. GNPs diameter was 10 nm verified by electronic microscopy and dynamic light scattering. Breast cancer cells were irradiated with doses between 0 and 6 Gy. This study also shows the cytotoxicity of GNPs and their effect on the cell survival without the irradiation component. In fact, viability of MCF-7 cells treated with different GNP concentrations (0, 5, 12.5, 25, and 50 mg l^− 1^) was measured and no significant effect was concluded, therefore the final results are triggered by the irradiation process. The uptake of GNPs by the cells was confirmed by experiment, there was a strong correlation between culture GNP concentration and the amount of gold uptake. After the first irradiation trials, they concluded that GNP concentrations of 25 and 50 mg l^− 1^ were capable of enhancing the radiation effects. Therefore, these two concentrations were used in further irradiations to study the cell survival after exposure to different doses ranging from 0 to 6 Gy. The results showed an increase in cellular radio-sensitivity after GNPs uptake, this effect was referred to as “dose enhancement”; we would rather define it as increased sensitivity since we have no direct measurements of the dose at the cellular level during irradiations.

Zheng and Chow (2017) presented another study using photon and electron beams for radiation therapy. MC simulations were used to assess the dose enhancement due to GNP presence at skin level. Concentrations of nanoparticles varied between 3 and 40 mg ml^− 1^. Megavoltage electron beams between 4 and 6 MeV of energy were used to irradiate skin target layers of thickness between 0.5 and 10 mm. Different types of nanoparticles were investigated, e.g. Au, Pt, I, Ag, and Fe_2_O_3_. Concentrations were varied between 3 and 40 mg ml^− 1^ and the DEF was calculated for each irradiation. Results showed a small enhancement for electron irradiations compared to those with photons. In fact, electrons are charged particles and they interact more frequently with the irradiated medium. Therefore, the presence of nanoparticles does not bring a noticeable enhancement to their mass-stopping power, which is the opposite for photons. The results showed an increase of the dose enhancement with the NP concentration and 4 MeV electrons had higher DEFs than those of 6 MeV. The irradiated target thickness also affected the enhancement with a positive correlation, and the maximum enhancement ratio reached for electrons was ~ 1.09 versus a ratio of 6.3 ratio for photons. Interestingly, in some electron irradiation cases the DEF was lower than 1.0 which means that the dose decreased with increasing NP concentration, this is more clearly seen for thin targets and high energy beams, mainly 6 MeV and for a target less than 2 mm thickness. In these particular cases, the NP can absorb part of the energy of the incident beam preventing further energy deposition in the biological medium.

Fathy et al. (2019) also showed experimental radiosensitizing effects of iron oxide and silica coated iron oxide nanoparticles on MCF7 breast cancer cells. Characterization of NP was carried out using dynamic light assessment and the results revealed an average size of ~ 18 nm for iron oxide NPs and ~ 164 nm for silica coated NP. Irradiations with 6 MeV electrons at different doses (0.5, 1, 2 and 4 Gy) showed an increased cell death rate when NPs were present in the cell medium. The cell death rate increased with NP concentration, which ranged between 5 and 80 µg ml^− 1^. Furthermore, the enhancement rate decreased with increasing irradiation dose. The maximum DEF was 2.13 using silica coated iron oxide with a concentration of 80 µg ml^− 1^ and for an irradiation dose of 0.5 Gy, while this factor was 1.64 when iron oxide particles were used with the same concentration and for the same irradiation dose. Although the observed effects were referred to as “dose enhancement”, in fact, results consisted of cell survival ratios and therefore the measurements are mainly “radiosensitization ratios”. The results of (Fathy et al. 2019) focused on the role of silica coating which improves the NP stability and increases the sought after radiosensitization.

Superparamagnetic iron oxide nanoparticles (SPIONs) were also used by Rahman et al. (2019) in a similar study investigating their radiosensitizing effects for electron beam irradiations and for brachytherapy. The interest of investigating SPIONs is that they are already used in MRI imaging as a contrast agent; therefore, their biocompatibility is already tested. The sensitization ratio increased with the SPIONs concentration in the medium. Irradiations were done with 6 and 12 MeV electrons and the effects were less important for the 12 MeV energies.

A detailed study by Gray et al. (2020) using the MCNP6 code (version 6.2) compared the dose enhancement effects of GNPs for ^192^Ir source irradiations with experimental results. The GNPs characterization showed a size of 29.8 nm diameter within 2.8 nm uncertainty and three mass concentrations were taken into account 0.28%, 0.56% and 0.77%. The experimental measurements used an in house-built irradiation and measurement device using the Burlin cavity principle. As far as the authors are aware, this is the only study that measures the physical dose enhancement experimentally. The comparisons between experiments and numerical simulations agreed within 2% and the DEFs were 1.018 ± 0.003, 1.031 ± 0.003, and 1.041 ± 0.003 respectively for the 3 GNP concentrations.

Electron oscillations in the gold structure can create a quasiparticle type called plasmon. A plasmon can be described as the quantified collective oscillation of free electron gas. The reason they are of interest for our topic is that they can vehicle energy within the irradiated solids. Therefore, the energy transmitted from incident particles to the electrons, in the gold or other materials nanometer volumes, can also create collective electron vibrations leading to plasmon creation. Plasmons can migrate within the GNPs and release their displaced energy or can also reach the surface of the nanometer sphere where they were created and provide enough energy to ionise an electron that would exit the nanoparticle. Therefore, taking plasmons into account it is expected that the number of electrons around nanoparticles increases. Knowing that these electrons are usually of low energies, they are expected to deposit their energy around the nanometer spheres. A study published by Sakata et al. (2016) showed the importance of the plasmon creation process in comparison with other energy loss processes in metals. In brief, the plasmon loss cross section is comparable to the electronic excitation cross section between 10 eV and 100 eV incident energies in gold. Another study by the same author (Sakata et al. 2018) showed MC based results of energy depositions around GNPs taking into account plasmon effects for photon irradiations. In conclusion, differences were observed in energy deposition patterns taking plasmons into account, especially for nanoscale simulations. These results were based on a comparison with Geant4 Penelope and Livermore condensed history models.

### Protons and heavy ions

Lin et al. (2014) carried out a study on the dose enhancement due to the presence of GNP, when the medium is irradiated with proton beams. The TOPAS MC toolkit was used for simulations. Clinical 6 MV and kilovoltage photon beams were used as references. It should be noted that these authors only account for secondary electrons produced by the protons in the GNP or WNP (equivalent water nanoparticle) when determining the dose around it. That is, the contribution from the primary particles to the dose is neglected. This approach may have introduced some bias in the DEF. They found that the DEF is independent of the proton energy, while for photons this dependence is strong. They also found that very close to the GNP, DEF is about 300, 400, and 6 for 150 kVp photons, 250 kV photons, and 10–150 MeV protons, respectively. The reader should notice that the DEF near the GNP surface depends weakly on the proton energy. At 10 μm, DEF is about 2000 for 150 kVp and 250 kVp photons and 13 for protons. They argue that photons produce much more energetic secondary electrons than protons, mainly by the photoelectric effect. As said above, protons have a mass much larger than that of electrons, so energy transfers are relatively small. Furthermore, the expected value for the energy transfer from protons and light ions to electrons is about 50 eV and quasi-independent from the incoming charged particle type and its energy. Another important finding of this work is that the activation of Auger electron production during simulations with incident protons leads to a dose increase of about 1.5%, even close enough to the GNP. However, for photons, Auger activation produces a 10-fold increase in the dose near the GNP. This shows that Auger electron emission is important for the dose enhancement for photon irradiation but not for protons. This group also applied this methodology for studying the DEF of GNP when small blood vessels are irradiated with protons, 6 MV photons, and x-rays (Lin et al. 2015). Results for DEF as a function of the incoming radiation quality were similar to those obtained in the former work.

Martínez-Rovira and Prezado (2015) have also studied the DEF for GNPs when irradiated with protons at the Bragg peak. Simulations were carried out with the GATE/Geant4 code. They also included Gd NP in the study. Unlike the work by Lin et al. (2014), this work accounts for the dose due to the primary particles, in addition to that from secondary particles produced by the GNP. Much lower DEF were obtained in this work, up to 1.7, while Lin et al. (2014) found DEF of 13–14. It could be argued that the primary proton beam is weakly perturbed by the GNP so that the overall impact on the dose distribution around this particle is weak. They also found that the influence of Auger electrons on DEF is relatively weak, going from about 1% near the GNP surface, to about 5% at 35 nm from the surface. This result may be explained by the fact that inner shell ionisations produced by charged particles are much less probable than those occurring in valence shells. Thus, the influence of Auger electrons on the DEF when irradiating with protons may be regarded as small. However, the work by Lin et al. (2014) showes that Auger electrons are important when photons are used as primary radiation. In this situation, Auger electrons can increase the dose in vicinity of the GNP up to 10 times. Photons can induce photoelectric effects in the GNP, which, unlike charged-particle ionisation, is more probable in inner shells, leading to fluorescence or Auger cascade production. Ahmad et al. (2016) also studied the impact of gold and other metal nanoparticles on the position of the Bragg peak, when a water solution is irradiated with 60 and 226 MeV protons. Nanoparticle concentrations ranged from 0.01 up to 6.5 mg ml^− 1^. In this work, dosimetry was carried out by Monte Carlo simulation and experimentally, using radiochromic films. They found DEFs determined by simulations of about 1.06 and 1.05 for proton energies of 60 MeV and 226 MeV, respectively. However, they reported experimental DEF of up to 1.21.

Cross sections for the interaction of incoming particles with gold is a key issue when studying the DEF. Hespeels et al. (2019) have recently studied the impact of proton inelastic cross sections for gold on the DEF and even on the Radiolysis Enhancement Factor (REF). They experimentally determined spectra for electrons emitted from 200 nm gold foils and compared them to those obtained by the Geant4 and TRAX MC codes. They also included the Binary Encounter Approximation into the Geant4 models. They found differences of about two orders of magnitude between the electron emission frequency (energy spectrum) between the BEA implemented in the TRAX code and the Bethe-Bloch approach included in Geant4, observing that the former code agrees better with experimental spectra than the latter. DEF and REF were determined for GNP with radii of 5 nm and 10 nm, using both methodologies for determining inelastic cross sections. It was observed that the original Bethe-Bloch approach in Geant4 leads to DEF lower than 1 near the GNP surface, while for the BEA cross sections, DEF is always greater than 1. The authors argue that the Beth-Bloc cross sections implemented in the original Geant4 code underestimate the emission of low energy electrons from the GNP.

Peukert et al. (2020a) carried out a study on the DEF and REF for GNPs irradiated by protons, using the Geant4 MC code. They studied the effect of 15 nm GNP on the dose distribution inside a cell model. GNPs were distributed into clusters of different sizes and numbers of GNPs (concentration), inside the nucleus or cytoplasm, and the impact on the dose distribution was studied. The model was irradiated with 5 MeV protons, for which DEF from 1.07 up to 1.19 were reported. They concluded that, due to the very short range of secondary electrons produced by proton impact, GNPs must be very close to the cell nucleus to have a sifting effect on the dose inside it. They found that DEF decreases with the energy of the incident protons, specifically when going from 10 MeV down to 5 MeV. At these energy levels, proton inelastic collisions may be divided into ionizations and excitations. As the incident proton energy decreases, the fraction of the proton energy invested in excitations increases, which means that fewer secondary electrons are produced, by ionization. This may be an explanation for the decrease of the DEF with the proton energy.

Torrisi (2015) reported DEF for a 100 MeV proton beam impacting on water, adipose tissue, and bone doped with GNP at a 10% concentration by weight, which can be regarded as very high. Calculations were made with the SRIM MC code (Ziegler et al. 2010). He defined the DEF due to the presence of GNP as the ratio between the energy loss per unit path length at the Bragg peak, with and without GNP for each medium. DEFs were in the 1.05–1.06 range for the tissue equivalent media studied and 100 MeV proton beam. This work also reported that, specifically for water, DEF increases linearly with the proton energy from about 1.04 at 50 MeV up to 1.08 at 200 MeV.

Jeynes et al. (2014) compared electron emission from GNPs irradiated with protons and x-rays. They studied this phenomenon using the Geant4 MC toolkit. They also determined survival curves for a cell line in vitro, irradiated with 3 MeV protons and 250 kVp x-rays, in presence of a free radical scavenger at different concentrations. They found that the scavenger plays a cell-protective role for x-ray irradiations in presence of GNPs. However, they did not observe any important effect of the scavenger in the same situation when irradiating with 3 MeV protons. They also found that the increase of electron emission due the presence of GNPs is almost negligible for protons, yet a very slow increase is observed as the proton energy increases, from 1 MeV up to 250 MeV. However, they observed a 3-fold increase in electron emission for x-rays. Another curious finding is that the electron spectrum shape is almost independent of the proton energy. This can be explained from the same behaviour observed for the proton ionisation cross section differential in the secondary electron energy (Bernal and Liendo 2006).

McKinnon et al. (2016) studied the dose enhancement around gold and two ceramic oxide (Ta_2_O_5_ and CeO_2_) nanoparticles, all with 100 nm diameter. They used the Geant4 MC toolkit to simulate the irradiation of a single NP with a proton beam with energies of 5 and 50 MeV. The DEF was defined as the ratio of the dose with and without the NP, within a cylinder with 2 μm diameter and 0.4 μm height, centred at the NP site. They found DEF of about 1.27 for GNP, almost doubling those for the oxide NP.

A comprehensive review on experimental and simulated DEF for metallic NP irradiated with ion beams was published by Peukert et al. (2018). The most important message from that review is that, while metallic NP produces less dose enhancement than x-ray beams, significant experimental cell radiosensitization could be explained by the increment in the production of reactive oxygen species, which is a matter outside the scope of this review.

**Fig. 3:**
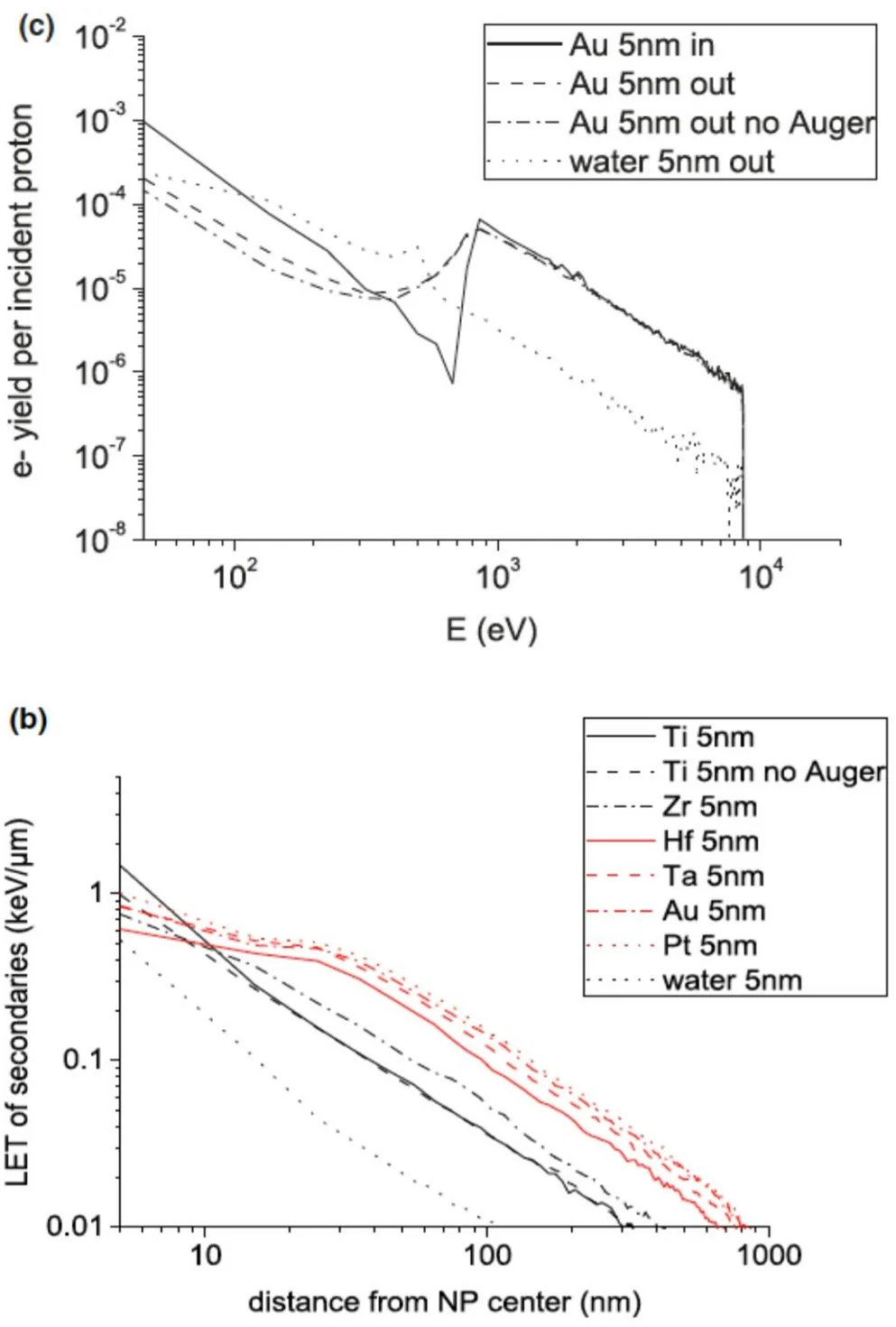
Secondary electron energy spectra (left) and their corresponding LET radial profiles (right) when 4 MeV protons impact on liquid water in presence of gold and other metallic NP. Figure permission use by Medical Physics, Wiley Periodicals LLC

Another study on the cell radiosensitization due the presence of several metallic NP, including gold, with sizes ranging from 5 to 50 nm was published by Heuskin et al. (2017). They used two geometrical setups in simulations: a single NP and a cylindrical cell model. The cell was modelled as two concentric cylinders, the smallest one representing the nucleus. No NP was allowed to be inside the nucleus. As far as we understand, the authors did not transport the incoming particles outside the NP after the interaction with it, so the perturbation of the primary spectrum in water due to the presence of the NP was not totally accounted for. Instead, they only studied the influence of NP on the secondary electron spectrum, specifically, how the NP influences the radial LET distribution for secondary electrons. For the 1.3 MeV proton beam, no impact of the Auger emission on secondary electron spectra was observed, but for the 4 MeV proton case, an increment of electron emission was found due to this process for energies up to about 500 eV. It would be expected that the very low energy protons cannot transfer enough energy for ionising Au core shells. Figure 3 (left panel) shows secondary electron energy spectra at the water and NP sphere surfaces. The presence of the NP leads to a softening of the secondary electron spectra below about 500 eV, when compared to that corresponding to liquid water. This can be attributed to a combination of the Auger process enhancement due to the presence of the GNP and the production of low energy electrons by ionisation and subsequent multiple inelastic scattering inside the NP. It should be noted that this spectrum softening occurs even after switching off the Auger emission during simulations. However, this spectrum is hardened above about 600 eV, probably due to the increment of electron production by ionisation of gold valence electrons, which can be regarded as quasi-free. In addition, electronic density in gold is larger than that of liquid water, which should lead to an increase of electronic stopping power and thus the production of more secondary electrons. The impact of changes observed in electron spectra due to the NP presence can be observed in the secondary electron LET profiles shown in the right panel of Fig. 3. There is an increment of electron LET at closer distances from the NP due to the softening of the electron spectra, as said just above, and electron ranges are longer due to an increase in the production of electrons above 600 eV, when compared to the pure liquid water situation. These important results show that the impact of the Auger emission enhancement due to the presence of GNP is restricted to a few ten nanometers from the NP. At longer distances, which should be more important for macroscopic dose distributions, the influence of GNP comes from the enhancement of secondary electrons production by ionisation.

Cho et al. (2016) studied the physical processes involved in the dose enhancement due to the presence of gold nanorods in a saline solution. Specifically, they made MC simulations for discriminating the effects of particle x-ray emission (PIXE), particle-induced gamma-ray emission (PIGE), and Auger electron production as well. In addition, they determined dose enhancement ratio experimentally, using radiochromic film dosimetry. They found that, although the dose enhancement ratio could be up to 17 in the vicinity of the nanorod, the overall enhancement on the macroscopic dose distribution is of the order of 0.1% at clinically relevant gold concentrations.

Both dose and chemical yield enhancements due to the presence of GNP when the medium is irradiated with x-rays, protons, and carbon ions, were investigated by Rudek et al. (2019). They used the TOPAS-nBio MC tool, which is based on the Geant4-DNA toolkit. The influence of GNP size, concentration, and clustering was investigated. The authors found that dose-enhancement ratios for 10 MeV protons and 100 MeV carbon ions were 1.003 and 1.004, respectively, at a 5% GNP concentration. Again, this study shows that the impact of GNPs on macroscopic dose distributions is very low for heavy charged particles. Results on chemical yields are not within the scope of this review.

The effect of 50 nm GNPs on cell killing after the irradiation of a cell culture with 200 MeV protons have been recently studied by Cunningham et al. (2021). They found that GNPs led to cell killing increases of 27% and 44%, at 2 Gy and 6 Gy absorbed doses, respectively. Another important result from this study is that no significant variation of cell killing enhancement due to GNPs was found as a function of depth, i.e. as a function of the proton LET. Enferadi et al. (2018) carried out also an in vitro study on the DEF and radiosensitivity increment induced by ultra-small functionalized GNP, with 1.8 nm diameter. They also investigated the GNP uptake by the cell culture, which was irradiated with x-rays, megavoltage photons, and protons. A DEF for protons of about 1.14 was reported.

Schuemann et al. (2016) reviewed the use of GNPs for dose enhancement in radiation therapy of cancer, including biological aspects such as cellular intake of GNPs. The authors briefly summarised physical key points related with dose enhancement, namely the relation between energy and type of incident particles and the associated physical interaction processes. In short, they showed that photons with energy in the 100–200 keV range lead to higher DEFs than those produced by megavoltage photons and heavy charged particles, like protons. The key physical interaction process is the photoelectric effect.

The MC code TRAX was used by Wälzlein et al. (2014) to study the dose enhancement around nanoparticles of several metals, including gold, the material in question in this review. Nanoparticles with radius of 2 nm and 22 nm were irradiated with 2.80 MeV and 300 MeV protons, and with 44 keV electrons. According to the models implemented in this MC code, the production of Auger electrons by the impact of 80 MeV protons in gold can be discarded above about 2 keV. Despite the fact that these protons can transfer up to about 174 keV to atomic electrons, ionisation of core shells is much less probable than in outer shells. Thus, Auger electrons with energies above 2 keV are virtually not observed. These authors reported the emission of 0.81 primary Auger electrons per 10 ionisations in gold. This number goes up to 0.91 if including secondary Auger emission, which involves ionisations from secondary electrons. Fluorescence photon emission is negligible since outer shells vacancies decay preferably by Auger emission. As reported in other similar works, GNP impacts on the dose distribution up to a few tens of nanometers. The energy of protons and electrons were chosen so that both particles travel at the same speed. In this situation, the inelastic cross section should be similar, based on the First-Born approximation. However, some slight differences have been observed due to target polarisation and other effects. These authors reported inelastic cross sections for protons slightly higher than electrons, but the Auger spectrum is also important for explaining the dose enhancement effect. This work reported that Auger electrons are more important for dose enhancement in the case of incident electrons than for protons. In short, dose enhancement due to GNP when irradiated with protons is restricted to very short distances from the metal surface. This shows that only very high GNP concentrations may lead to important increments of the macroscopic dose.

Mohseni et al. (2020) extensively studied the dose enhancement phenomenon for GNP. Photons, electrons, protons and alpha particles were used as primary particles for irradiating GNP with radii ranging from 20 nm to 150 nm, at a fixed concentration of 10 mg g^− 1^ in liquid water. These particles were distributed inside a cell geometrical model, including a nucleus and a cytoplasm. Dose distributions were determined using the Geant4-DNA package. This work also shows that DEF is close to unity for protons and alpha particles. This is explained by the low energy of secondary electrons emitted by ionisation due to the impact of heavy charged particles, as well as for low energy of Auger electrons. This leads to dose enhancement only very close to the GNP, no more than a few tens of nm. They also reported single and double strand break yields with and without the presence of GNP and showed no significant differences between them. However, the authors did not report the involved uncertainties in damage yield, thus a rigorous analysis cannot be made.

DEFs for protons and carbon and oxygen ions were recently studied by Fuss et al. (2020). Nanoparticle size ranged from 1.2 up to 22 nm. The interaction between incident particles and water with nanoparticles was simulated with the TRAX MC code. The authors used two simulation setups: one, where incoming particles are forced to first interact with the gold or equivalent water nanoparticle, and only secondary electrons were allowed to escape from the nanoparticle, and the other where the full interaction of the primary particle with both the nanoparticle and the surrounding water is accounted for. It should be remembered that the first approach is similar to that used in (Lin et al. 2014). For the first setup, they found a nearly spherically symmetric dose distribution around the nanoparticle, but the reader must keep in mind that the directional preference of the incoming primary particle has been removed artificially in this situation, since only secondary electrons were accounted for. An interesting result from this work, when only electrons are accounted for, is that a very small, isolated GNP of the order of a few nanometers behaves as a single source of secondary electrons. As the diameter increases, the resulting dose distribution around the GNP is composed by two wings, like being produced by two independent sources. This behaviour is related to the self-absorption within the GNP. As the diameter increases, electrons produced near the centre of the GNP have more difficulty for escaping from it. In addition, the DEF as a function of the distance from the isolated GNP center, shows a peak at about 0.9 μm and 1 μm for the 2 nm and 7 nm GNP diameters, respectively. For the 22 nm GNP, no peak appears, just a plateau from about 0.8 μm onwards. Curiously, the structure of these DEF curves is nearly independent of the incoming particle type and energy, including the position of the DEF peaks. The highest DEF at the peak were found for the smallest GNP, up to about 20 (accounted for the dose only from secondary electrons), however, for longer distances the three GNP produce DEF that converge into the 1–12 range. Again, these very large DEFs for protons are only obtained accounting for the secondary electrons only.

An interesting work by Parishan et al. (2020) investigated the effect of a magnetic field on the dose distribution around gold and iron oxide nanoparticles. Simulations were performed with the TOPAS toolkit and found no important effects of the magnetic field on the dose distribution. It seems that the field is not strong enough to perturb the electron transport around the nanoparticle.

Akhdar et al. (2022) simulated the secondary electrons produced around a nanoshell GNP cluster irradiated by proton beams using Geant4 code. In the simulation, protons of 100 MeV were sampled from a 2D squared area source of 5 μm x 5 μm, located 1 m away from a water brain phantom with dimensions of 6 cm x6 cm x 6.7 cm. The whole apparatus was positioned within an air-filled volume of 120 cm x 120 cm x 120 cm. Simulations showed an increased secondary electron production at distances of 10–100 nm from the surface of all nanospheres. The DEF decreased to less than 1 at distances larger than 100 nm from the surface of GNP. The authors concluded that the clustering of GNP leads to an important localized dose enhancement.

Rajabpour et al. (2022) performed simulations of a single GNP with 50 nm diameter placed in a cubic water phantom (10 cm x 10 cm x 10 cm) irradiated by 5, 50, and 150 MeV proton beams, using Geant4 (v10.07.p02) code. A total of 10^9^ incident protons were simulated to satisfy an acceptable statistical uncertainty of less than 1%. Radial dose distribution, DEF, G-value, and radiolysis enhancement factor (REF) were calculated. Authors found that the secondary electron spectrum using the Penelope model is 30% higher at energies between 250 eV and 1 keV than using the Livermore model. However, the G-value and REF were not affected by the choice of physical interaction models. It is interesting to mention that in the range of 6 nm from the surface of GNP, DEF using the Penelope model is 15% higher than using the Livermore model; and after 6 nm, the DEF using the latter was slightly 7% higher than that using the former. Overall, for 5 MeV protons, DEFs ranged from 1.4 to 11 at the distance of about 1 μm and decreased sharply and increased again at a distance of 30 μm. For 50 and 150 MeV proton irradiations, DEF increased from 1.5 to 14 until the maximum range of the secondary particles.

In recent works (Tabbakh 2024, 2026), Tabbakh showed that the dose enhancement effects produced by protons are due to the increase of their LET due to the presence of the GNPs. He devised a simulation with GNPs incorporated into cells at a given weight concentration. This high-density material disrupts the incoming particles in such a way that their stopping power is importantly increased when compared with that observed in a pure water medium. Thus, the impact of GNP on the dose may be observed far away from GNP, although secondary electrons only lead to a very localised dose enhancement, up to a few tens nanometers.

## Discussion

Radiation therapeutic beams can be classified according to the primary particles, e.g. uncharged particles like photons and charged particles such as electrons, protons and heavy ions. This classification is fundamental because the primary particles determine how the beams interact with GNPs and how these particles influence the dose distribution. Dose enhancement originates from the increment of radiation absorption from GNPs when compared to that in liquid water or biological tissues. GNPs lead to an absorption increase for both photon and charged particle beams, but the physical processes involved for each kind of radiation are relatively different, and therefore also the way GNPs impact on dose distribution.

In setup I, DEF depends on the energy of the incident beam, the distance from the GNP and its size. In setups II and III, gold concentration is an additional influence parameter. It is important to look at the nature of secondary particles produced after primary radiation absorption by the GNPs. Removal of inner shell electrons is a key process when dealing with the dose enhancement produced by GNPs. The nature of the primary particle strongly influences cross sections for this electron process. Primary photons predominantly undergo this process via photoelectric absorption, while charged particles do it by impact ionisation. It should be noted that the photoelectric attenuation coefficient increases with decreasing photon energy (~ 1/*E*^3^). On the one hand, the photoelectric effect is more probable for atomic inner shells than for the outer ones. On the other hand, charged particles ionize preferentially valence shells. This means that primary photons should lead to higher dose enhancements due to increased photoelectric absorption and the subsequent x-ray fluorescence or Auger electron emissions, when compared with incident heavy charged particles and even megavoltage photons. Consequently, the overall dose distribution is strongly influenced by the generation, transport, and local energy deposition of these secondary radiations. Gold is a high-Z element that preferentially de-excites after inner-shell ionisation by emitting characteristic x-rays when compared with Auger electron emissions. These fluorescent photons and Auger electrons are produced at similar energies for a given target, therefore it is expected that photons affect dose distribution at larger distances from GNPs than Auger electrons, since the former have longer ranges than the latter. However, these fluorescent photons originating in a GNP can produce a cascade of photoelectrons in the same GNP or in the nearby GNPs; these fluorescent photons and photoelectrons can escape from a GNP and deliver dose in distal water farther away from this GNP. Furthermore, macroscopic dose enhancement depends on the range of secondary particles produced by radiation-GNP interactions and GNP concentration. For all these reasons, primary photons with energies near the gold *K*- or *M*-shell binding energies should be the best option for dose enhancement when compared with primary charged particles.

One key remaining question is the influence of GNP size on interaction cross sections. It is well known that at such small dimensions surface effects can arise. Up to our knowledge, all MC codes use cross sections determined for bulk material which include gold. Therefore, the research community should address this problem to improve MC simulations in this context.

The reviewed literature indicates that DEF trends are largely consistent with established radiation interaction physics which was shown in Fig. 2. In particular, higher DEF values are frequently reported for photon irradiation, especially in the keV energy range, where the contribution of photoelectric absorption is enhanced compared to MV photon beams. For charged particles (protons, heavy ions, and electrons), the reported DEF values tend to be lower on average; however, their magnitude and relative ordering are highly dependent on multiple factors, including particle energy, material composition, nanoparticle concentration, spatial distribution, and the specific definition of DEF used. Several studies suggest a comparatively weak dependence of DEF on incident energy for protons, heavy ions, and electrons within certain energy ranges. In the following, we give a brief summary of DEFs for setups I, II, and III, in liquid water irradiated by different incident beams.

Early simulations showed greater values of DEF in the vicinity of the GNP surface in the setup I, irradiated by kVp x-rays or keV photons. However, in many of these simulations, the photon beam was assumed to impinge a single GNP with a beam of the same GNP size. In this setup I, lateral charged particle equilibrium cannot be achieved. It is manifest that the dose enhancement in the near proximity of the GNP is considerably attributed to Auger electrons. Low-energy electrons released from the interaction of x-ray fluorescent photons with the same GNP superpose the dose enhancement to a similar extent. Several authors investigated systematically the dose enhancement for a single GNP and the results of DEF values can represent the average values for photons reviewed in the literature published to date. For a single GNP with diameters from 10 nm to 100 nm, located at 2–20 cm depth in water phantom, the maximal DEFs range from 50 to 20 for irradiations by 82–88 keV monoenergetic photons and from 120 to 56 irradiated by 16–22 keV monoenergetic photons. The optimal energies and GNP sizes for a maximum DEF were near 20 keV to 30 keV, and 100 nm of GNP, respectively. In distant regions from the GNP, photoelectrons of the prime photoelectric absorption process dominate the dose enhancement. The steep increase and fall of the DEF as a function of the distance from the GNP surface depends mainly on the number of escaping electrons from the GNP. However, for MV photon beams, DEFs are relatively low, of about 5.8–1.8 depending on the flattering filter.

There are some simulations on clustered GNPs, setup II, which reported DEFs ranging from 5 up to 10 for keV photons, but there is no data for MV beams. In this setup, DEF would depend on the GNPs concentration and the corresponding range of secondary radiation emitted by them.

Many other simulations were performed for gold diluted in water at some concentration (Setup III), for which DEF values were found to be around 1.01–1.10. For a 16 Au mg g^− 1^ homogenized gold concentration in a tumour model at 2 cm depth, the average DEF is between 1.2 and 3.0 for energy ranging from 20 to 100 keV. An earlier simulation showed a slightly greater DEF of 3.8 with a concentration of 18 Au mg g^− 1^ in a tumour model at 0.075 cm depth.

There is limited simulation work focused on single GNPs (Setup I) irradiated by electron beams. However, the limited data showed that for a single GNP irradiated by electron beams, DEFs are in the range of 2.5–12. DEF is not significantly dependent on the electron energy. For GNP distributed in tumor models (Setup II), DEFs are often limited to 1.03. DEFs scored in nucleus and cytoplasm will be decreased to about 1.0 for the GNPs distributed in cells. For electron beams, DEF mainly depends on the size and concentration of GNPs in the media.

For heavy charged particles, such as protons or heavier ions, DEFs have been reported between 1.0 and 2.0 DEF values show little dependence on the incident proton energy. For a single GNP irradiated by proton beams in water (Setup I), a maximum DEF is reported about 14 (Lin et al. 2014) and 20 (Fuss et al. 2020). For gold aqueous solution (Setup II), the DEF is about 2.03.

The reader should keep in mind that very high DEFs found near the GNP irradiated by keV-energy photons may not be traduced into a large impact on the macroscopic dose distribution. As mentioned before, this impact depends on the volumetric density of GNPs, their corresponding DEF, and the distance at which this DEF is observed. This distance is strongly influenced by the range of the secondary particles emitted by the GNP. For instance, very high DEFs are observed very close to the GNP surface, mainly determined by very short-range Auger electrons. Since their range is in the order of a few nanometers, their impact on the macroscopic dose distribution is negligible, unless prohibitely high GNP concentrations could be reached. Thus, DEFs at several tens of micrometers from the GNP are more important for the macroscopic impact of these particles on the macroscopic dose distribution.

One important question is the possible shortening of particle penetration into the medium due to the presence of GNPs. It could be expected that a significant macroscopic dose enhancement should lead to a shorter penetration of the beam in question. Heavy charged particles may present a stronger effect on the penetration when compared with photons, since charged particles show a well-defined range and photon beam dose distributions decrease smoothly and without a defined range. We have included in this review a recent paper showing that LET increasing due to the presence of a GNP distribution is the main factor responsible for the macroscopic dose enhancement when dealing with heavy charged particles, such as protons. This factor is much more important than the localized dose enhancement around each GNP and can also explain the dose increase at long distances from the GNP.

In conclusion, it is evident that photon beams, mainly low energy ones, provide the highest DEF among all the particles covered by this review. For an overview on the distribution of DEF, please refer to Table S2 in the Supplementary Information.

The impact of GNP on the macroscopic or even microscopic, yet not nanometric, dose distributions can be obtained by a convolution of the dose kernel around a single nanoparticle and the spatial distribution of nanoparticles within the medium. Throughout this review, it has become evident that the range of dose enhancement around GNPs is primarily determined by the type of incident radiation. Photons, in particular, produce a broader dose distribution around individual GNPs compared to charged particles. The physical basis for this behavior has been elaboratelydiscussed in Sect. S2.2 in the Supplementary Information. Consequently, there is a clear interrelationship between GNP concentration, the type of incident radiation, and the resulting impact of nanoparticles on both macroscopic and microscopic dose distributions.

An open question is how the size of GNPs influences radiation-gold interaction cross sections. To the best of our knowledge, all MC codes currently used in this field rely on cross sections derived for bulk gold. However, it would be scientifically valuable to investigate whether GNP size affects these cross sections. At the nanoscale, quantum or surface effects may emerge that alter interaction probabilities (Emfietzoglou and Incerti 2020). Additionally, GNP size impacts self-absorption of emitted radiation, with larger nanoparticles tending to absorb a greater fraction of the secondary radiation they generate.

## Conclusions and perspectives

GNPs have been widely investigated and regarded as radiosensitizers in nanoparticle-enhanced radiotherapy. Both in vitro and in vivo experimental investigations have demonstrated observable enhanced biological effects when GNPs are irradiated with various types of radiation. MC radiation transport codes have served as the gold standard tool for simulating physical dose deposition and the subsequent generation of chemical species at the microscopic scale to correlate enhanced radiation doses with biological responses. However, these simulations vary significantly due to differences in radiation types and energies, as well as in the characteristics of the GNPs, used in different MC codes, including the sizes, shapes, concentrations and spatial distribution in irradiation medium or in cellular and molecular environment.

MC codes used by different modellers for the GNP dose enhancement are generally categorised into two models with three setups, namely, condensed history (setup I) and mixed condensed history (setup II) models, and track structure (setup III) models. The special resolution of these two class codes is different because these codes used different physical simulation strategies and different physical interaction cross sections. In addition to MC codes, deterministic codes were also used in this research field for accelerating calculation times and for a low statistical uncertainty. The difference of simulation methods and the various MC classes need further quality assurance of these codes used. All the aforementioned aspects may reveal the phenomena that the simulated physical radiation doses presented different values with a large uncertainty. This topical review restricts the review of the energy deposition and DEFs simulated using MC codes and some deterministic codes. The reviewed information on energy depositions and DEFs may provide essential information for setting up a dose-response relationship in GNP-aided radiotherapy and for further quantifications of the enhanced chemical and biology effects.

From this review, it is obvious to see that at comparable GNP sizes and concentrations photon beams produce greater DEF values than proton, heavy ion, and electron beams. For a single GNP irradiated by photon beams, the DEF can reach from 20 up to 120 nearby a GNP, depending on the GNP size and photon energy. DEF for keV photons is about 9 times greater than for MeV photons at maximum. Same for a single GNP, the DEF values for electrons, protons, and heavy ions are much smaller, ranging around 1.0 to 14 at maximum. For gold concentration in liquid water or in tumours, the DEF for these various radiation beams showed a similar value around 1.01–1.10. For photons beams, under a high concentration of gold in liquid water, a maximum DEF value of 3.0 can be reached. Despite the low DEF values, further MC simulations estimating DEF of GNPs potentially used in clinical Linac MV and proton beam treatment setting should be continued. For theoretical radiobiological research, some medical physics associations and international committees may make a benchmark for quantification of DEF for a single GNP irradiated by kVp photon, proton, and electron beams in a fixed clinical setting. These DEF values or energy spectra could be used as kernels to generate DEF for multiple GNPs from macroscale conditions, using mathematical integral methods, such as the Green’s functions.

Beyond the reviewed results in the physical dose enhancement factors, we recommend several perspectives in the GNP-aided radiotherapy for the future simulation work. First, there is rare work on the dose enhancement for GNPs which are coated with ligands and antibodies, etc. This simulation can provide the energy absorption by the molecules bound to GNPs and provide a realistic dose to the tumour. Second, since new radiation therapy modalities, like FLASH and microbeam radiotherapy, are introduced to the preclinical and clinical use, a strategy is needed for simulating the dose enhancement with GNPs in this ultra-high dose rate radiotherapy delivering very high doses in a very short time scale. Third, extended studies are needed on dose enhancement factors for application of GNPs in targeted radionuclide therapy with new emerging radiopharmaceuticals, especially alpha- and beta-emitters. If the energies of photons and electrons of the targeted radiopharmaceuticals are greater than the ionization potential of the targeting molecules, a greater density of low-energy electrons may be generated. Fourth, a quantification is needed of surface effects and the auto-ionization process of low energy electrons generated near GNPs. Surface excitation of electrons near the surface region of GNPs are associated with surface plasmons that can further enhance the release of low energy electrons (10–30 eV). While this effect may have a small impact on the dose enhancement, it is not negligible. Furthermore, these very low energy electrons react with the surrounding medium by resonances and can be captured by molecules. This capture leads to formation of transient anion molecules that can decay through auto-ionizations or dissociations (Zheng and Sanche 2020). These processes can induce bond cleavage and thereby contribute to biomolecular damage. Fifth, results calculated by deterministic codes which combine the Green’s functions should be compared to those obtained with MC codes, to check if the timesaving deterministic codes can be used for simulating different clinical settings. Sixth, the DEF is a relative value to demonstrate the enhanced dose effect; however, this DEF does not exist in real radiation therapy. The absolute energy deposited and absorbed dose in the target region may be used as an indicator for radiobiological effects.

At last, our review on the enhancement of physical radiation dose in GNPs can encourage other peers to explore and review the complementary enhanced chemical and biological effects in GNP-aided radiotherapy. In addition, we want to encourage radiobiologists for extensive review work on enhanced biological effects obtained from in vitro cellular experiments and in vivo small animal investigations. Merging knowledge on physical, chemical, and biological mechanisms is key to advancing and translating GNP-enhanced radiotherapy from the laboratory to the clinic.

## Supplementary Information

Below is the link to the electronic supplementary material.

Supplementary Material 1

## Data Availability

No datasets were generated or analysed during the current study.
