# Supplementary material for: Range of dose enhancement factor for gold nanoparticles in radiotherapy using Monte Carlo simulations
Source: Radiat Environ Biophys. 2026 Aug 8;65(4):999–1027. doi: 10.1007/s00411-026-01245-4 (PMC13687369; doi:10.1007/s00411-026-01245-4)
Supplement: Supplementary file 1 [file 411_2026_1245_MOESM1_ESM.pdf]

Range of dose enhancement factor for gold nanoparticles in radiotherapy using Monte Carlo simulations

Wei Bo Li[1,2,*], Mario A Bernal[3,*] and Ziad Francis[4]

[1]Institute of Radiation Medicine, Helmholtz Zentrum München, German Research Center for Environmental Health (GmbH), Ingolstädter Landstraße 1, 85764 Neuherberg, Germany.

[2]Federal Office for Radiation Protection (BfS), Ingolstädter Landstraße 1, 85764 Oberschleißheim, Germany.

[3]Instituto de Fisica Gleb Wataghin, Universidade Estadual de Campinas, Campinas 13083-859, SP, Brazil.

[4]Saint Joseph University of Beirut, Research Unit Mathematics and Modeling, Beirut 1004 2020, Lebanon.

*Corresponding authors

Wei Bo Li

Federal Office for Radiation Protection (BfS)

Ingolstädter Landstraße 1

85764 Oberschleißheim

Germany

E-mail: wli@bfs.de

ORCID: 0000-0002-9802-4400

Mario A. Bernal

Instituto de Física Gleb Wataghin

Universidade Estadual de Campinas

Campinas 13083-859, SP

Brazil.

E-Mail: mabernal@unicamp.br

ORCID: 0000-0002-8305-5298

Supplementary information

S2.1 Interactions of radiations with liquid water and gold

The main physical processes on the radiation interactions with liquid water and gold for photons, electrons, protons, and heavy ions were described in the following.

S2.1.1 Photons

Five types of interactions of photons with matter are commonly considered in radiation physics (Evans 1955; Marmier and Sheldon 1969; Attix 1986): Compton scattering, photoelectric effect, electron–positron pair production, Rayleigh scattering, and photonuclear interactions. Rayleigh scattering is an elastic photon interaction and is only important at low energies (below 100 keV), especially for the high-Z materials, such as gold. This interaction is only important for scattering of photons, not for energy absorption. Photoelectric effect is the dominant interaction process for photons of energies up to around 500 keV for high-Z atoms (such as Z = 60–80) and the cross section strongly depends on the atomic number Z of the target atom and the photon energy E, like $\sigma \propto (Z/E)^n$, with $n \cong 3$–4. Compton scattering becomes the dominant interaction process for high-Z atoms as the photon energy becomes greater than 500 keV. The associated atomic cross section is proportional to Z. Electron-positron pair production happens for photon energy larger than 1.022 MeV and its cross section is proportional to Z^2, but it is only important at energies of several MeV. Calculated cross sections for the processes in liquid water and gold are available from the NIST/XCOM database (Seltzer 1987). This database can be used in conjunction with other databases for quantifying the energy deposition in the investigated materials. In Fig. S1 (A) shows the total mass attenuation coefficient of interactions of photons with liquid water and gold, (B) shows the ratio of the mass attenuation coefficients between gold and liquid water, (C) shows the mass energy-absorption coefficient of interactions of photons with liquid water and gold and (D) shows the ratio of the mass energy-absorption coefficients between gold and liquid water.

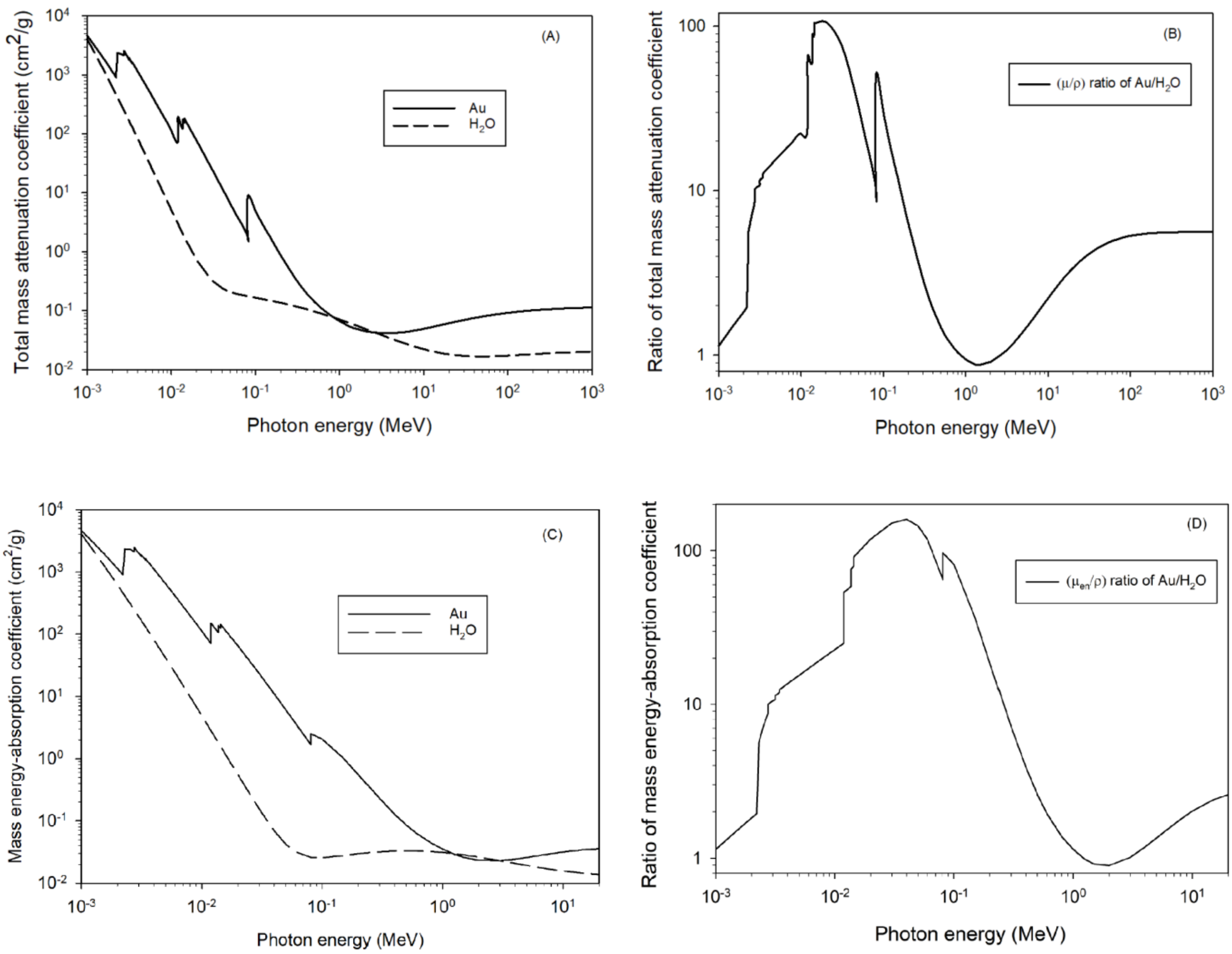

Fig. S1. (A) Total mass attenuation coefficients of the interaction of photons with gold (Au) and liquid water (H_2O). (B) Ratio of total mass attenuation coefficients of Au and H_2O. (C) Mass energy-absorption coefficients of the interaction of photons with Au and H_2O. (D) Ratio of mass energy-absorption coefficient of Au and H_2O. The ratios depicted in figures (B) and (D) were calculated based on the interpolated mass energy-absorption coefficients retrieved from NIST/XCOM.

S2.1.2 Electrons

Electrons are the dominant secondary particles produced by ionising radiation through irradiated media. They are also abundantly produced in GNPs after photon collisions. Therefore, the importance of detailing and understanding their interactions assumes that most energy deposition points are created by secondary electrons. On top of their abundance in irradiated media, their interaction cross section increases at low energies, showing a maximum in the so-called Massy peak, at about 125 eV. Interactions of low energy electrons include inelastic collisions, e.g., ionisation and excitation. Elastic scattering

is also frequent at low energies while bremsstrahlung emission becomes increasingly important at higher energies with an energy loss contribution of around 5% for energies above ~2 MeV. Depending on the interest and on the irradiated target, we might also consider vibrational and rotational excitations for very low energies. These processes become important at extremely low energies (<7 eV) where electrons are below the liquid water ionisation and excitation thresholds. For applications in radiobiology and DNA damage quantification, sub-excitation processes (Platzman 1955) become important since all the secondary electrons will undergo slow energy loss at the end of their tracks. Furthermore, photons can undergo pair creation collisions and ejected positrons can go through the annihilation process.

Incident electrons interact with electrons bound to target atoms and molecules of the medium. The energy is transferred through the mutual interaction of electric fields of charged particles, and the total interaction cross section is directly related to the electron density in the target medium. As a general approach, it is possible to calculate the so-called microscopic cross section per target electron and normalise the obtained value using the electron density to calculate the inverse mean free path. The microscopic cross section for inelastic collisions can be calculated using the first-Born approximation (FBA) for high energies, which means energies much higher than the kinetic energy of the target orbiting electrons. The FBA becomes inaccurate for low energies and can be corrected using empirical formulas to adjust the resulting cross section and stopping power to experimental data, or reference data such as the ICRU stopping cross section values (ICRU 1984, 2014). The inelastic collisions of electrons, electronic excitation and ionisation are dominant throughout the whole energy range between the target's ionisation threshold and few MeV. Below the ionisation and electronic excitation thresholds, respectively ~10 eV and ~8 eV in water, interactions are dominated by molecular vibration, rotation, and translation modes. The elastic collisions are present through a wide energy range and become increasingly important at low energies especially below ionisation and excitation thresholds. Electron attachment to target molecules can also contribute to energy deposition by electrons at the end of their track in water, this process is most probable around ~8 eV, the related cross section is represented as a narrow peak around an average energy value. Elastic scattering does not affect the electron's kinetic energy but only its direction.

S2.1.3 Light and heavy ions

Light and heavy (Z>2) ions are charged particles, like electrons, so they interact with matter through electromagnetic processes, namely electronic excitations, ionizations, and elastic scattering from atoms. However, unlike electrons, ions are composed by baryons so they can also undergo nuclear reactions mediated by the nuclear force. They can also interact through the weak force, but this is not an important mechanism during atomic collisions. Another important difference between ions and electrons is that the former are much heavier than the latter. Thus, at the energies used in medical applications, ions travel

at speeds for which relativistic effects may be discarded in most situations, mainly along the so-called Bragg peak of the energy deposition curve. Therefore, the Bremsstrahlung process can be disregarded at such low speeds. Ions do not suffer from interchange effects during electronic processes, as electrons do because the former are distinguishable particles when compared with atomic electrons, the most important target during atomic collisions.

Large ion mass, when compared to that of electrons, is responsible for the relatively low energy transfer during ionisation processes. On the one hand, an electron can transfer up to a half of its energy to another electron. On the other hand, an ion can transfer to an electron a fraction of its energy of the order of m/M, where m and M are the electron and ion masses, respectively. This means that ions would transfer their energy through a very large sequence of individual collisions, during which a huge number of very low energy electrons are produced. For instance, the average energy of electrons emitted by ion impact on water is about 50 eV, which is a result that depends weakly on the heavy projectile type. Another important process influenced by the large ion mass is the elastic scattering. Unlike electrons, ions scatter at much lower angles so most of their trajectories are done along a virtual straight line. For kinematical reasons, these trajectories become more tortuous when energies go below about a few tens keV u^{-1}. At relatively low energies, ~keV u^{-1}, ions can undergo elastic multiple scattering along short paths. It should be noted that during the elastic scattering, the incoming ion interacts with the whole target atom or molecule. This means that the ion-target interaction potential can be described as a nucleus-nucleus potential with screening provided by the bound electrons.

At relatively high impact energies, ionisation is the most frequent inelastic interaction, while at low energies, of the order of a few tens keV u^{-1}, excitation gains relevance. Excitations have a more limited impact on DNA damage than ionisation, since during excitations no secondary electrons are produced. However, both processes can lead to subsequent dissociative decay of the water molecule, producing reactive chemical species that can also induce DNA damage.

There are some differences between light and heavy ions, from the interaction with matter point of view. Heavy ions can have higher nuclear charges, which can lead to much more complex processes, such as double ionisation and multiple electron capture. These processes are rarely observed for light ions, like protons and alpha particles. In addition, heavy ions produce more nuclear reactions than light ions, including those leading to important neutron production. These reactions are mediated by the strong nuclear force, which has a range of the order of 1 fm. In short, nuclei can interact with each other at very small impact parameters, with the mediation of the strong force, leading to nuclear elastic or inelastic collisions.

S2.2 Mechanism of energy and dose enhancement

Dose enhancement due to the presence of GNP depends on the primary radiation type and energy, which determine the physical processes involved in the radiation-gold interaction process. In the following, in addition to the secondary electrons emitted from the interaction of primary particles with gold, we introduce the de-excitation processes by which further secondary electrons, e.g. Auger electrons, are generated and x-ray fluorescence is produced.

S2.2.1 De-excitation processes

Both electromagnetic radiation and charged particles can produce ionizations in inner atom shells. There are also nuclear processes that can create such vacancies, like internal conversion and electron capture. As an electron is ejected from an inner atomic shell by any of these processes, such as the photoelectric effect for photon beams, the vacancy formed by these mechanisms leave the atom electronically excited, the excited atoms relax to their ground state through two competitive processes, radiative (x-ray fluorescence) or non-radiative (Auger effect) transition by a relative probability (Evans 1955). The probability of the emission of fluorescent photons occurring is called fluorescence yield, ω and 1-ω will be the probability of the Auger effect occurring. The atomic number of the target atom and the energy of the incident beam determine the balance between these two mechanisms. Fig. 4 (left) shows Auger and fluorescence yields as a function of the target atom atomic number (Z). Excited low-Z atoms tend to decay by Auger emission, while the high-Z atoms are more likely to emit characteristic x-rays. Both photons and Auger electrons are emitted with energies up to the excitation energy of the atom where the ionisation was produced. In high-Z atoms, a complex cascade may occur, and several photons or Auger electrons may be produced.

The balance between these two processes influences the dose distribution around the small material volumes, where radiation equilibrium may not be established. Fluorescent photons carry their energy farther from the emission point, while Auger electrons travel very short distances, commonly of the order of a few tens nanometers in liquid water, when emitted from the high-Z nanoparticles.

As indicated by the vertical dashed line shown in Fig. S2 (left), the excited gold atoms are more likely to decay by x-ray emission with 96% if electrons in the *K*-shell is ejected, which means the incident photon energy should be greater than 80.7 keV, the binding energy of the *K*-shell (see Table 1). If the incident photon energy is greater than 14.4 keV, a vacancy in the *L*-shell can be produced, for which the probability of x-ray emission is only 33%. The Auger emission spectrum for gold is very complex, due to the complexity of its electronic structure, as in any high-Z material. If the incident photon energy is further reduced to 2.2 to 3.5 keV, the probability of x-ray emission from *M*-shell is very low and the probability of Auger electron emission is much dominant, 97.5%. From Fig. 4 (right), the main emission lines of Auger electrons are observed at 69 eV and

2 keV (Childs and Hedberg 1995). This means that Auger electrons emitted from gold are mostly *MNN* transition (for notation of Auger electrons, see Fig. S3) and have a relative low energy, up to 2 keV. Since water is formed by low-Z atoms, de-excitation mainly occurs by the emission of Auger electrons from the oxygen *K*-shell, with energy of about 500 eV.

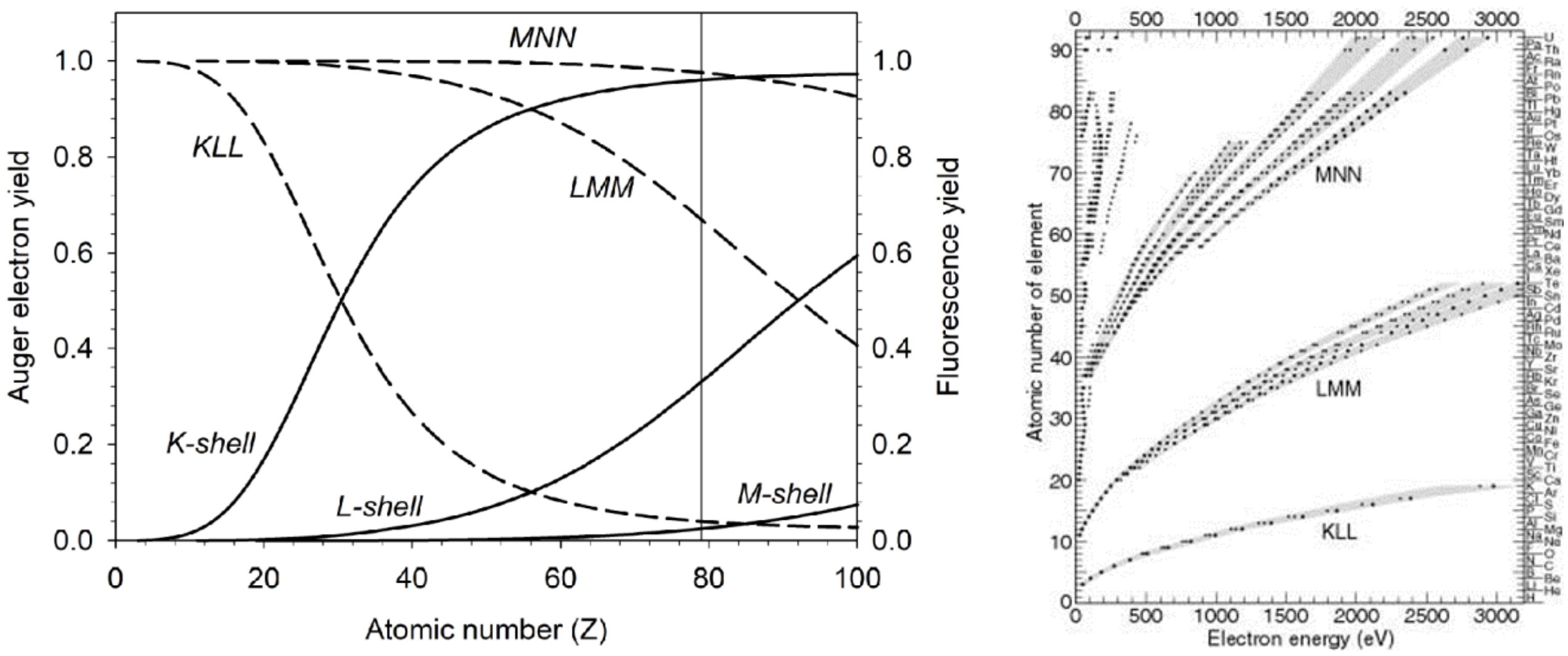

A **B**

Fig. S2**.** (A) X-ray fluorescence yield (right y-axis) in *K*-shell, *L*-shell and *M*-shell (solid line) for and Auger electron emission yield (left y-axis) of *KLL*, *LMM* and *MNN* (dashed line) of the atom as a function of the number of Z of materials. The solid vertical line indicates the yields for gold (Z=79). (B) Energies of Auger electrons released by transitions *XYZ* of different materials with atomic number Z (Childs and Hedberg 1995). For gold, with Z=79, Auger electrons are released by the transition *MNN*, with energies ranging from 1500 to 2000 eV. Some Auger electrons with lower energies can also be seen from transitions *LMM*.

Table S1. Electron binding energies and x-ray fluorescence emission lines in gold. Data were retrieved from the Appendix 2 by S. Hagström et al. in Siegbahn (1968).

Au											
Shell	Binding energy (eV)	$K\alpha_1$	$K\alpha_2$	$K\beta_1$	$K\beta_3$	Shell	$L\alpha_1$	$L\alpha_2$	$L\beta_1$	$L\beta_2$	$L\gamma_1$
K	80723	68.8	67.0	78.0	77.6	*L*	9.7	9.6	11.4	11.6	13.4
L_1	14353	54.45	52.64	63.63	63.22						
L_2	13733	55.07	53.26	64.25	63.84						

L_3	11918	56.89	55.07	66.06	65.66					
M_1	3425	65.38	63.57	74.56	74.15	6.29	6.20	8.02	8.16	9.96
M_2	3149	65.66	63.84	74.83	74.43	6.56	6.48	8.29	8.44	10.23
M_3	2743	66.06	64.25	75.24	74.83	6.97	6.88	8.70	8.84	10.64
M_4	2291	66.51	64.70	75.69	75.28	7.42	7.34	9.15	9.29	11.09
M_5	2206	66.60	64.78	75.77	75.37	7.51	7.42	9.24	9.38	11.18
N_1	760	68.05	66.23	77.22	76.81	8.95	8.87	10.68	10.82	12.62
N_2	645	68.16	66.35	77.34	76.93	9.07	8.98	10.80	10.94	12.74

As shown in Fig. S2, a spectroscopic notation is used to describe Auger electron emission. For instance, a KL_2L_3 electron was emitted from the L_3 subshell after another electron had jumped from the L_2 sub-shell down to the K-shell vacancy of the atom in question. The special case in which the electronic transition takes place within two sub-shells of the same shell is known as the Coster-Kronig process, for instance the transition $L_1L_2M_1$.

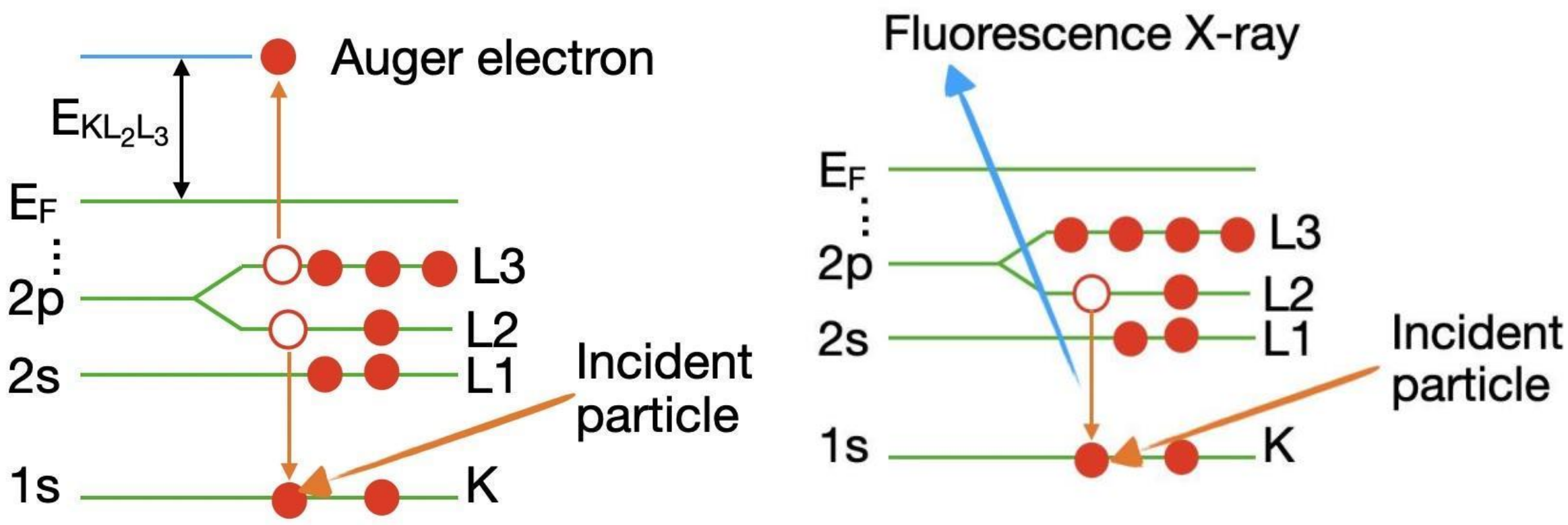

Fig. S3. Example of the Auger emission process (KL_2L_3 transition) and fluorescence x-ray emission ($K\alpha_1$). Initially, a vacancy is generated in the K-shell. This can occur by a photoelectric effect, impact ionization, electron captured by an ion, or by nuclear processes such as electron capture and electron conversion. This vacancy is then filled by an electron with higher energy, from the L_2 sub-shell in this example. Finally, an electron from the L_3 sub-shell is emitted by the atom. E_f is the Fermi energy level of the metal. For detail refer to Evans (1955) and Attix (1986).

S2.2.1.1 Contribution from x-ray fluorescent photoelectrons and Auger electrons to dose enhancement

As aforementioned, the dose enhancement around high-Z GNPs results from two main physical processes: photoelectrons emitted by photoelectric absorption of photons from GNP atoms and the Auger electrons produced by the ensuing atomic de-excitation processes. X-ray fluorescent photons produced from the target atom de-excitation can further produce photoelectrons and Auger electrons within the GNP. The kinetic energy of the photoelectron is the difference between the incident photon energy and the binding energy of the electron in the atomic shell. After photoelectric absorption in the *K*- and *L*-shells, fluorescence x-rays are generated and they could be reabsorbed within the same or in other nearby nanoparticles. Of course, a fluorescent photon emitted by gold only can produce a photoelectric effect if its energy is greater than the target electron binding energy. This can make further photoelectric effects in higher shells due to the substantially higher cross-sections at these energies. If the GNP is very small, these photons may escape without further interaction and they will contribute to the dose farther away in the liquid water, resulting in a dose enhancement in the surroundings. However, at larger GNP sizes, the same GNP absorbs a greater fraction of these fluorescent photons, and the subsequent photoelectrons are energetic enough to escape a relatively large GNP. For example, when $L\alpha$, $L\beta$, and $L\gamma$ fluorescent emissions ionise the *M*-shell, the resulting photoelectrons have energies between 6.2-11.2 keV (table 1), with respective ranges in gold of 170-440 nm (Meesungnoen et al. 2002). Ionisation of the *N*-shell yields even more energetic photoelectrons with a minimum energy of 8.9 keV. When $K\alpha$ and $K\beta$ fluorescent emissions are absorbed by the *L* and higher shells the resulting photoelectrons have a minimum energy of 52.6 keV with 5.5 µm range in gold. This conclusion is valid even for very low energy fluorescent photons, at the $M\alpha$ emissions. At these energies (∼2 keV) larger GNPs have increasingly greater photoabsorption probabilities, resulting in *N*-shell ionizations, which yield photoelectrons at ∼1.5 keV (Table S1) with an escape depth of 16 nm (Sajo and Zygmanski 2020). Note that ionizations of higher shells (*N* and up) by fluorescence photons do not contribute to the dose enhancement via Auger but they do via photoelectron emission. This is because, for these events, the Auger electron energies are generally below 300 eV and they are absorbed within the GNP. Fluorescence x-ray, therefore, give rise to a cascade of photoelectrons originating in the higher shells with sufficient energy to escape from the GNP, similar to the Auger electrons above 1 keV, having dense ionisation tracks. Because the fluorescence yield from the *K*-shell is about 96% and from the *L*-shell it is ∼37% for gold (Bambynek et al. 1972; Cullen et al. 1997), the combined contribution of the photoelectrons produced by primary photons and x-ray fluorescence photons to the dose enhancement, is comparable to that of Auger electrons.

S2.2.2 Plasmons

As irradiated by x-rays, GNPs can de-excite via decay of surface plasmons, which are collective oscillations of free electrons on the surface of nanoparticles (Kittel 1966), this was also mentioned by Kuncic and Lacombe (2018). Following an excitation event such as a low-energy Coulomb collision, thermal equilibrium is eventually restored through electron-phonon interactions (Kittel 1987). Equilibrium can be also recovered through recombination of thermalized electrons, whereas sub-excitation electrons can also undergo dissociative electron attachment to surrounding water and other biomolecules, causing further radiation-induced damage (Boudaïffa et al. 2000). Furthermore, metallic clusters, such as GNPs (3–40 atoms) irradiated by 1 MeV protons, may induce electronic excitations in the GNP atoms due to the ionisation of from outer shells due to excitation of plasmons (Verkhovtsev et al. 2015a, b) with theoretical and quantum chemistry calculations. It implies that collective excitation followed by plasmon damping makes a non-negligible contribution to low energy electron emission in the vicinity of GNPs.

S2.2.3 Charge transfer

Emission of multiple low energy Auger electrons may result in a highly charged nanoparticle in the surface of smaller GNPs. Such kind of positively charged nanoparticle is highly reactive and may extract electrons from water and other biomolecules in the neighbouring environment. This phenomenon is so called neutralisation process and it can drive energy transfers and charge transfers in a very small-scale environment (Wagenknecht and Gray 2006; May and Kühn 2011; Stumpf et al. 2013). These ultra-fast relaxation processes may ionise surrounding molecules and produce every low energy electron, which can further amplify radiation damage beyond that predicted by Auger decay processes (Li 2006; Howell 2008; Ndlebe et al. 2010; Ndlebe et al. 2012).

Table S2: Summary of references on calculation of DEF (until 2022). The references are referred to the main text.

Study	Source	GNPs, single/multi particulate/compound	GNP size, diameter	Geometry	MC code	Primary particles	Energy deposition	DEF
Radiation beam: photons								
Cho (2005)	140 kVp x-rays, 4 and 6 MV photon beams, and ^{192}Ir gamma rays.	Compound, 7 mg Au g^{-1} in tumour	-	tumour	EGSnrc/EGS4, MCNP5	$4x10^6$	-	1.007 to 5.601
Carter *et al* (2007)	100 keV x-rays	GNP, single	3 nm	DNA	Own code	Flux=2.31x10^{10}/cm^2/s for 60 s	10^{-5} eV/nm^3	250% for SSBs chemical effects included
Zhang *et al* (2009)	^{192}Ir HDR parallel and 4π-isotropic beam, 15 mm above the beam	10^{13} GNPs cm^{-3}, concentration of 9.7%	Sphere, 100 nm	water phantom	Geant4.8.1.p01	10^9 photons	1.8-5.6x10^{-5} Gy	128%
Jones *et al* (2010)	^{125}I, ^{103}Pd, ^{169}Yb, ^{192}Ir as brachytherapy. 50 kVp and 6 MV x rays target 5–8 cm depth in water phantom	7 mg Au g^{-1}	-	Tumour 3×3×3 cm^3	EGSnrc, OREC	0.5-50×10^6	relative	10 to 1000 and 10 for 6 MV photons using kernels
Leung *et al* (2011)	50 kVp, 250 kVp, ^{60}Co, and 6 MV,	GNP single	2, 50, and 100 nm	Source-target 1 mm, width of the photon beam equal to the diameter of the GNP	Geant4	2.5×10^8	Yes, relative	-
Lechtman *et al* (2011)	^{103}Pd, ^{125}I as brachytherapy, ^{169}Yb, ^{192}Ir. 300 kVp and 6 MV x-rays	GNP, single and concentration	1.9, 5, 30 and 100 nm	1 cm tissue depth for brachytherapy and 5 cm 300 kVp and 6 MV (phase-space files)	MCNP5 and PENELOPE-2018	10^9-2×10^{10}	In range	-
McMahon *et al* (2011a)	20-150 keVand 160 kVp x-ray	GNP, single	2-50 nm	GNP at the centre of a cube of water with sides of 200 mm	Geant4 v4.9.3 with Geant4-DNA	1×10^8 - 2×10^9	25 eV in the first 1 nm shell. 10^5, 4×10^4 and 3×10^2 Gy in the first 1 nm shell	-
Detappe *et al* (2013)	6 MV photon beam	30 mg ml^{-1}	100 nm	Tumour 10×10×2 μm	EGSnrc and DOSXYZnrc, analytical calculation, Auger	-	-	EDF in endothelial cell: 1.2, to 4.5

					electrons are excluded			
Cai *et al* (2013)	10-100 keV, 100 kVp x-ray and ^{125}I and ^{103}Pd brachytherapy	GNP, 2×10^4-8×10^5 GNP per cell	5, 30 and 50 nm	GNPs in extracellular space, on the cell surface, in the cytoplasm, or in the nucleus	MCNP5	5×10^9–2×10^{10}	In cell	DEF in nucleus: 3, DEF in cell: 2
Douglass *et al* (2013)	80 kVp, 6 MV	Single GNP cluster, gold foil	400 nm, 300 nm layer	GNPs in the cytoplasm, gold layer around nucleus	Geant4	10^6	yes	55 in nucleus
Lechtman *et al* (2013)	300 kVp	GNP, single and concentration	30 nm	GNPs in cell culture media, photon penetrating 4 mm tissue, phase-space file	MCNP5, PENELOPE-2008		Not shown	Sensitive enhancement factor: 1.29, 1.20 and 1.07
Zygmanski *et al* (2013)	11 keV–1 MeV photon beams, 50 kVp x-ray.	GNP, single and multiple in cluster	100 nm	Parameters in nm–µm geometry: source-to-GNP distance, beam size, and GNP size	Geant4, CEPXS/ONEDANT	10^8 - 2×10^9	Yes, not shown	DEF for single: 640 (beam size =GNP size)<br>DEF for single:10-75<br>DEF for cluster: 36-46<br>DEF for shell: 2-100
Garnica-Garza (2013)	50, 80, 100, 150 and 200 keV, 220 kVp x-ray	Single	2 and 100 nm	A GNP is embedded in a semi-infinite water	PENELOPE	-	Lineal energy distribution	-
Li *et al* (2014)	60, 80, 100 and 150 kVp x-ray	Single and gold concentration	2, 15, 50, 100 and 200 nm	Source to GNP distance: 1 mm, beam size = GNP size	PENELOPE, Geant4, EGSnrc	10^7 - 10^8	Yes	DEF single: 500-1000<br>DEF for gold concentration: 2-5
Lin *et al* (2014)	50, 100, 150, 200 and 250 keV, 150 and 250 kVp x-ray; 6 MV Linac	single	50 nm	GNPs embedded at various depths in a water phantom, phase space, beam size=GNP size	TOPAS	10^9 (gold) - 2×10^{10} (water)	Radial dose	800-1000 for keV photons<br>40 for kVp x-ray<br>20 for MV photons
Casta *et al* (2015b)								
Casta *et al* (2015a)	60 keV	single	20, 40 and 100 nm	Casta et al 2014 model	Own, Geant4	10^7	Emission of secondary &	-

							Auger electrons and x-ray fluorescence	
Xie *et al* (2015)	60, 80, 100, 150 and 200 kVp	Single and multiple	14, 50, 74 and 100 nm	Within cell	PARTRAC	10^8 - $2x10^{10}$	yes	10000 for single 5-18 for multiple
(Kirkby and Ghasroddashti 2015)	90, 125, 200 and 300 keV, ^{60}Co and 6 MV wff and fff	Single and multiple	13 nm	GNPs in membrane of mitochondria	PENELOPE	10^9	yes	1.1-2.3 in mitochondria 8.2 for local voxel
Incerti *et al* (2016a)	10 and 100 keV	Single	10 and 100 nm	Beam matching GNP diameter	Geant4 version 10.2 beta	$2x10^{10}$	Emission of secondary & Auger electrons	-
Koger and Kirkby (2016)	Photons from 20 keV to 6 MeV; 100, 200, and 300 kVp x-rays	15 mg Au g^{-1} in tumour, 0.8 mg Au/g in normal tissue	2 and 20 nm	GNPs in tumour with sizes 1, 2, 3 and 4 cm; Arc radiation therapy	PENELOPE	10^7	Dose distribution	3
Ferrero *et al* (2017)	160 kVp, 6 MV, and 15 MV	$5x10^5$ GNPs per tumour cell	2 nm	Phase space	Geant4 version 10.1 and Geant4-DNA extension	$4x10^8$ - $2x10^{10}$	Radial dose distribution	-
(Strigari et al. 2017)	160 kVp, 6 MV, and 15 MV	$5x10^5$ GNPs per tumour cell	2 nm	Phase space	Geant4 version 10.1 and Geant4-DNA extension	$4x10^8$ - $2x10^{10}$	Radial dose distribution	-
Kirkby *et al* (2017)	10, 20, 40, 50, 60, 80, 85, 100, 200 and 500 keV, 1 and 10 MeV	GNP cluster, 9 Au mg g^{-1}	50 nm	GNPs cluster inside a water sphere with 1000 nm diameter	PENELOPE-2011	10^9	Radial energy	Effects of cluster GNPs on a GNP irradiated
Koger and Kirkby (2017)	1 keV-200 keV	GNP cluster,10 and 20 mg Au g^{-1} in water	10, 20, and 50 nm	500-1000 GNPs with and without coatings (5, 10, and 20 nm, placed in cavities of side lengths 0.8-4 μm	PENELOPE	10^7	Radial energy as a function of coatings	2.5
Sung and Schuemann (2018)	50, 100, 150, 200 and 250 kVp, 6 MV	Single and multiple	15 nm	GNPs in cell and extracellular, phase spaces	TOPAS-nBio	-	-	In effect enhancement of dose ratios to tumour and healthy tissue

Rabus *et al* (2019)	50 and 100 kVp x-rays	Single	50 and 100 nm	Distance source and GNP: 10 μm. beam size=GNP size+10 nm	Using data Geant4, Geant4-DNA	10^8	yes	350 narrow beam 19 correction
Engels *et al* (2020)	150 kVp x-ray	Single and multiple	15 and 100 nm	GNP placed in 6 mm depth in a liquid water phantom, Beam with lateral size 10μm	Geant4, Geant4-DNA	10^{11}	Yes	22 for 15 nm GNP 48 for 100 nm GNP CH model overestimated DEF than TS
Vlastou *et al* (2020)	Review							
Li *et al* (2020a, 2020b)	50 and 100 kVp x-rays	Single	50 and 100 nm	Distance source and GNP: 10 μm. beam size=GNP size+10 nm	PENELOPE, Gean4 and geant4NDA, TOPAS and TOPAS-nBio, MCNP6, PARTRAC, MDM, NASIC	10^7-10^8	Yes	500 in nm 40 in μm
Poignant *et al* (2021)	20, 50, 70 and 90 keV	Single	10, 25, 50 and 100 nm	GNP in volume with side size of 30-360 μm	MDM, NanOx	-	specific energy distribution	Using probability enhancement ratio (PER): 95 at 5 nm from surface of GNP and 1.5 at 200 nm
Simiele *et al* (2021)	80 and 150 keV Inverse Compton beams	Single and gold concentration, 10 Au mg g^{-1} in brain model	-	GNP doping in planning target volume (PTV)	Egs_brachy user code of EGSnrc	-	Yes, dose profile	1.06 for IC 80 keV
Kolyvanova *et al* (2021)	30 to 300 kVp x-rays	Gold concentration, 10 Au mg ml^{-1}	-	Spherical tumour model with radius =2 cm	Geant4	2×10^8	-	1.09 - 2.36
Melo-Bernal *et al* (2021)	10, 30, 100 keV, 662 keV (^{137}Cs), 150 and 250 kVp x-rays	-	2, 10, 20 and 50 nm	circular beam matching GNP size	TOPAS	8×10^8	Radial dose	-
Kim *et al* (2021)	150 kVp x-rays	10^6 – 10^7 GNPs/cell	1.9 nm	Cell nucleus	Geant4/Geant4-DNA	-	Lineal energy and	Sensitizer enhancement

							absorbed dose	ratio: 1.41 and 1.29
	Radiation beam: radionuclides							
McLaughlin *et al* (2013)	^{225}Ac, ^{221}Fr	Gold coated lanthanide phosphate NP		Female BALB/c mouse model	-	-	-	-
Gholami *et al* (2019)	^{213}Bi, ^{223}Ra, ^{90}Y, ^{177}Lu, ^{67}Cu, ^{64}Cu and ^{89}Zr	SPIONs not GNP	-	-	-	-	-	1.2
Trujillo-Nolasco *et al* (2021)	Review	GNPs conjugated to ^{211}At for targeted alpha therapy see references [105,106] within review						
Silva *et al* (2021)	^{67}Ga and ^{177}Lu, ^{125}I	Small gold core carrying DOTA chelators and substance P (SP) peptides GNP-SP and GNP-SPTyr8	4 nm	Glioblastoma (GBM) cells: U87, T98G and U373. Model a matrix of nine cells. The central cell is modelled as composed by the cytoplasm and nucleus. The nucleus radius is 5 µm and the entire cell diameter is 25 µm.	-	-	-	DEF: 6-2 for x-rays and 1.5 for electron emission of ^{177}Lu
	Synchrotrons							
Rahman *et al* (2012)	Synchrotron radiation source of mean energy 125 keV; Superficial therapy machine (SXRT) 80 kV x-ray beams; 6 MV x-rays and 6 MeV electron beams from Linac	GNPs doped in normoxic type polyacrylamide gel (nPAG), 0.197 mg ml^{-1},	1.9 nm	Polymer gel	-	-	-	DEF: 1.76 for SXRT kilovoltage x-ray beam; 1.64 for synchrotron source; 1.37 for an electron beam; 1.14 for high energy x-ray beams.
Rahman et al (2014)	synchrotron-generated monoenergetic X-rays 30, 40, 50, 60, 70, 81, and 100 keV	GNPs in Bovine aortic endothelial cell suspension, 0.197 mg ml^{-1}.	1.9 nm	Bovine aortic endothelial cells	Ratio of mass energy absorption	-	-	Cell radiosensitivity enhancement

					coefficients obtained			was indicated by DEF: 3.47
Gagliardi *et al* (2020)	Clinical superficial x-ray: 150 kVp; Pre-clinical synchrotron beamline mean: 95.3 keV x-ray; Clinical Linac: 6 MV x-ray	GNPs incorporated in radiochromic polyurethane dosimeter PRESAGE	5 and 50 nm	-	-	-	-	DEF for 5 and 50 nm GNPs: 1.13 and 1.16 for 150 kV x-ray; 1.16 and 1.22 for 95.3 keV x-ray; 1.03 and 1.05 for 6 MV x-ray
	Microbeam							
Ghita *et al* (2017)	278 eV microbeam	Gold concentration	-	Cell model with nucleus	Geant4	-	-	No dose enhancement
Radiation beam: electrons								
Zheng *et al* (2008)	60 keV electron beams	Gold films and GNPs	ca. 5 nm	Plasmid DNA	-	-	-	Probability formation of SSBs/DSBs increases 2.5
Chang *et al* (2008)	6 MeV clinical electron beams	GNPs, 180 μm ml^{-1}	13 nm	B16F10 melanoma tumour-bearing Mice	-	-	-	Apoptotic cells increase 75%
Zhang *et al* (2009)	^{192}Ir with 380 keV photons	GNPs, spherical, 10^{13} cm^{-3}	100 nm		Geant4 v4.8.1	10^9 photon	Dose: 1.0-6.0x10^{-5} (Gy?) at 13 mm	1.2 to 1.3 around source. 1.15 at 17 mm
Chow *et al* (2012)	50 keV, 250 keV; 1 MV and 4 MV electron beams	Single GNP, spherical	2, 50 and 100 nm	Beam size = diameter of GNP	Geant4	2.5×10^8	Secondary electron energy self-absorption: 20% to 80%	Energy deposition ratio: 2.5–12
Rogers (2013)	Comments on Chow *et al* (2012)							
Koger and Kirkby (2016)	150 eV to 2 MeV	Bulk mixture or explicit GNP, 5, 10, 15, 20, 30 mg Au g^{-1} tissue	2, 10, 20, 50, 100 nm	Tumour with radius 1, 2, 3, 4 cm	PENELOPE-2011E	10^7	energy deposition was scored not shown	Dose ratio: 0.97-1.03
Mousavi *et al* (2017)	6 MeV electron beams	3 and 30 μM	50 nm					
(Farahani *et al* 2019)								

Mehrnia *et al* (2017)	4 MeV electron beams	GNPs, 5, 12.5, 25 and 50 mg l^{-1}	10 nm	Breast cancer cells				
Zheng and Chow (2017)	4 and 6 MV electron beams	GNP 3 and 40 mg l^{-1}		skin				1.09
Fathy *et al* (2019)	6 MV electron beams	Oxide NPs and silica coated NP, 5 and 80 μg ml^{-1}	18 nm and 164 nm	MCF7 breast cancer cells				Cell survival ratio: 1.64-2.13
Rahman *et al* (2019)	-Experiment. 6 and 12 MeV electron beams; ^{192}Ir 390 keV photons	SPIONs, concentration of 1, 2 and 3 mMol/L	15 nm	-	-	-	-	Sensitization enhancement ratio (SER): 1.5
Gray *et al* (2020)	^{192}Ir	Mass conc. 0.28%, 0.56% and 0.77%	30 nm	Cell level	MCNP6.2	10^9		1.018-1.031
Sakata *et al* (2016)	Discrete electrons transport model, plasmon cross section	-	-	-	Geant4-DNA with electron down to 10 eV; ELSEPA			-
Sakata *et al* (2018)	1, 10 and 100 keV electron beams	Single	20, 60, and 100 nm	Electron beams irradiate hemispherical surface of GNP	Geant4-DNA with discrete model: electron 10 eV	10^8-5×10^8	Radial absorbed dose	10 at < 100 μm 200-300 at > 100 μm
Radiation beam: protons and heavy ions								
Lin *et al* (2014)	10, 25, 50, 100, and 150 MeV proton beams	Single	50 nm	Beam size = GNP size	TOPAS	10^9-2×10^{10}	Radial dose	14
Martinez-Rovira and Prezado (2015)	200 MeV proton beams	Single	4 to 50 nm	Beams irradiate cubic water phantom 40 cm, phase space, shell 2 μm to 0.25 mm	Geant4-DNA	3×10^9	Radial dose	1.7
Incerti *et al* (2016b)	1 and 10 MeV proton beams	Single	10 and 100 nm		Geant4 version 10.2 beta	10^9-2×10^{10}	yes	
Hespeels *et al* (2019)	2, 5 and 10 MeV proton beams	Single, with 2 nm thick PPAA	2.5 and 3.8 nm	protons impinge target before 1 nm	TRAX and Geant4	10^7	Coating reduces electron yield at low energy, <500 eV	-
Peukert *et al* (2020)	0.5, 2, 5, 10, 15, 25, 50 MeV proton beams	Single	2 to 200 nm	Beam size= GNP size, phase space	Geant4 v10.4	10^5, 10^6	Dose vs GNP size	9-11

Torrisi (2015)	60 MeV proton beams	GNPs in ocular melanoma model, 10 Au mg ml^{-1}	5-20 nm	Model 3 cm behind eye lens	-	-	Proton energy in tumor increased 50%	-
Ahmad *et al* (2016)	60 and 226 MeV proton beams	Nano-film, Gold concentration, 0.01, 0.1 and 6.5 Au mg ml^{-1}	50 nm thickness	Nano-film in between Bragg peak, beam diameter 7 and 2.7 mm	Geant4 v10.0p02	8×10^6	Dose distribution from nano-film	1.07
Jeynes *et al* (2014)	1-250 MeV proton beams	GNP single, 30–100 GNPs clustered in vesicle (150 to 350 nm)	50 nm	64 μm^3 cubic water phantom	Geant4.10.p01, CLHEP 2.1.3.1 and ROOT 5.28.00	10^6		-
McKinnon *et al* (2016)	5 and 50 MeV proton beams	Ceramic oxide NPs Ta_2O_5 and CeO_2	100 nm	NPs in tumour	Geant4-DNA		-	1.27
Peukert *et al* (2018)	Review							
Heuskin *et al* (2017)	1.3 and 4 MeV proton beams; 5.3 MeV alpha.	Ti, Zr, Hf, Ta, Au, Pt nanoparticle, single	5–50 nm	NPs loaded cell model	Geant4 v10.3	10^6	LET distribution	-
Cho *et al* (2016)	-	GNP and gold nanorod (GNR), single and in mixed concentration with water	13 nm diameter, 44 nm length	phase space	TOPAS	10^9	Percentage depth dose	14.5 for single GNR, Negligible for uniform mixture of water and gold (0.03 wt.%)
Rudek *et al* (2019)	1-100 MeV protons; 1, 10 and 100 MeV carbon ions	GNP cluster and gold concentration, Au wt. 5% concentration	10 nm	GNPs in 0.9 µm diameter cell	TOPAS-nBio	-	-	1.03 for protons and heavy ions in cytoplasm
Cunningham *et al* (2021)	-Experiment		50 nm					
Enferadi *et al* (2018)	-Experiment	GNPs coated	1.8 nm					
Schuemann *et al* (2016)	-Review							
Walzlein *et al* (2014)	2, 80 and 300 MeV proton beams; 44 keV electron beams	GNP and Fe, Ag, Gd, Pt nanoparticles	2.4–44 nm	proton beam size=GNP size	TRAX	10^6	Radial dose	2
Mohseni *et al* (2020)	1-250 MeV proton beams: 1-2 MeV alpha: 50 keV-15 MeV electron beams	Gold concentration, 10 Au mg g^{-1} around nucleus	20–150 nm for photons and electrons	Planar source with 6×6 µm irradiating from 6 µm.	Geant4, Geant4-DNA	10^7	-	1.0

Fuss *et al* (2020a)	15, 80 MeV proton beams, 3–204 MeV u^{-1} for ^{12}C and ^{16}O ion,	GNP, single, 'isolated' and 'embedded'	2.4–44 nm	infinitesimal ion beam emitted by a point-like source	TRAX	10^5 - $5x10^8$	Radial dose	20
Parishan *et al* (2020)	70 and 150 MeV proton beams	GNP, SPION, single	40 and 100 nm	Source 3x3cm, distance source to target 20 cm, phase spaces	TOPAS	$2.5x10^7$, 3×10^9	Radial dose distribution	DEF=5 Magnetic DEF=1.04
Akhdar *et al* (2022)	100 MeV	Nanoshell GNP cluster		5 µm x 5 µm square source 1 m away	Geant4		Secondary electrons	ca. 1.0
Rajabpour *et al* (2022)	5, 50, and 150 MeV	Single GNP	50 nm		Geant4	10^9	Secondary electron spectrum, radial dose distribution, G-value, DEF	DEF=1.4 – 14

References

Attix FH (1986) Introduction to Radiological Physics and Dosimetry. John Wiley & Sons, Inc., New York Chichester Brisbane Toronto Singapore

Bambynek W, Crasemann B, Fink RW, Freund HU, Mark H, Swift CD, Price RE, Rao PV (1972) X-Ray Fluorescence Yields, Auger, and Coster-Kronig Transition Probabilities. Rev Mod Phys 44 (4):716-813. doi:10.1103/RevModPhys.44.716

Boudaïffa B, Cloutier P, Hunting D, Huels MA, Sanche L (2000) Resonant formation of DNA strand breaks by low-energy (3 to 20 eV) electrons. Science 287 (5458):1658-1660. doi:10.1126/science.287.5458.1658

Childs KD, Hedberg CL (1995) Handbook of Auger Electron Spectroscopy: A Book of Reference Data for Identification and Interpretation in Auger Electron Spectroscopy. 3rd. edn. Physical Electronics, Incorporation, Eden Prairie, Minn

Cullen DE, Hubbell JH, Kissel L (1997) EPDL97: The evaluated photo data library `97 version.

Evans RD (1955) The Atomic Nucleus. International Series in Pure & Applied Physics. McGraw-Hill, New York

Howell RW (2008) Auger processes in the 21st century. International Journal of Radiation Biology 84 (12):959-975. doi:10.1080/09553000802395527

ICRU (1984) Stopping Powers for Electrons and Positrons. ICRU Report 37. International Commission on Radiation Units and Measurements, Bethesda (MD)

ICRU (2014) Key Data For Ionizing-Radiation Dosimetry: Measurement Standards and Applications. ICRU Report 90. International Commission on Radiation Units and Measurements, Bethesda (MD)

Kittel C (1966) Introduction to Solid State Physics. Third edn. John Wiley & Sons, Inc., New York, London, Sydney

Kittel C (1987) Quantum theory of solids. 2nd rev. print edn. Wiley, New York

Kuncic Z, Lacombe S (2018) Nanoparticle radio-enhancement: principles, progress and application to cancer treatment. Physics in medicine and biology 63 (2):02TR01. doi:10.1088/1361-6560/aa99ce

Li WB (2006) Calculation of DNA strand breakage by neutralisation effect after 125I decays in a synthetic oligodeoxynucleotide using charge transfer theory. Radiat Prot Dosimetry 122 (1-4):89-94. doi:10.1093/rpd/ncl416

Marmier P, Sheldon E (1969) Physics of Nuclei and Particles, vol 1. Academic Press, New York

May V, Kühn O (2011) Charge and Energy Transfer Dynamics in Molecular Systems. 1st. edn. Wiley,

Meesungnoen J, Jay-Gerin J-P, Filali-Mouhim A, Mankhetkorn S (2002) Low-energy electron penetration range in liquid water. Radiation Research 158 (5):657-660. doi:10.1667/0033-7587(2002)158[0657:leepri]2.0.co;2

Ndlebe T, Neumann RD, Panyutin IG (2012) Study of charge transport mechanisms in ^{125}I-induced DNA damage at various temperatures. International Journal of Radiation Biology 88 (12):941-947. doi:10.3109/09553002.2012.697645

Ndlebe T, Panyutin I, Neumann R (2010) Analysis of the Contribution of Charge Transport in Iodine-125-Induced DNA Damage. Radiation Research 173 (1):98-109. doi:10.1667/RR1865.1

Platzman RL (1955) Subexcitation electrons. Radiation Research 2 (1):1-7

Sajo E, Zygmanski P (2020) The role of Auger electrons versus photoelectrons in nanoparticle dose enhancement. In: Sajo E, Zygmanski P (eds) Nanoparticle Enhanced Radiation Therapy: Principles, methods and applications. IOP Publishing, pp 1-1-1-13

Seltzer S (1987) XCOM-Photon Cross Sections Database, NIST Standard Reference Database 8. National Institute of Standards and Technology. doi:10.18434/T48G6X

Siegbahn K (1968) Alpha-, Beta- and Gamma-Ray Spectroscopy, vol 1. Noth-Holland/American Elsevier,

Stumpf V, Kolorenč P, Gokhberg K, Cederbaum LS (2013) Efficient pathway to neutralization of multiply charged ions produced in Auger processes. Physical Review Letters 110 (25):258302. doi:10.1103/PhysRevLett.110.258302

Verkhovtsev AV, Korol AV, Solov'yov AV (2015a) Electron Production by Sensitizing Gold Nanoparticles Irradiated by Fast Ions. J Phys Chem C 119 (20):11000-11013. doi:10.1021/jp511419n

Verkhovtsev AV, Korol AV, Solov'yov AV (2015b) Revealing the Mechanism of the Low-Energy Electron Yield Enhancement from Sensitizing Nanoparticles. Physical Review Letters 114 (6):063401. doi:10.1103/PhysRevLett.114.063401

Wagenknecht H-A (2005) Charge Transfer in DNA: From Mechanism to Application. Wiley-VCH, Weinheim. DOI:10.1002/3527606629
